# IFN-γ-independent immune markers of *Mycobacterium tuberculosis* exposure

**DOI:** 10.1038/s41591-019-0441-3

**Published:** 2019-05-20

**Authors:** Lenette L. Lu, Malisa T. Smith, Krystle K. Q. Yu, Corinne Luedemann, Todd J. Suscovich, Patricia S. Grace, Adam Cain, Wen Han Yu, Tanya R. McKitrick, Douglas Lauffenburger, Richard D. Cummings, Harriet Mayanja-Kizza, Thomas R. Hawn, W. Henry Boom, Catherine M. Stein, Sarah M. Fortune, Chetan Seshadri, Galit Alter

**Affiliations:** 1 Department of Immunology and Infectious Diseases, Harvard TH Chan School of Public Health, Boston, MA USA; 2 Ragon Institute of MGH, MIT and Harvard, Cambridge, MA USA; 3 Department of Medicine, University of Washington, Seattle, WA USA; 4 Department of Biological Engineering, MIT, Cambridge, MA USA; 5 Department of Surgery, Beth Israel Deaconess Medical Center, Harvard Medical School, Boston, MA USA; 6 Department of Medicine, Makerere University, Kampala, Uganda; 7 Department of Medicine, Case Western Reserve University and Univ. Hospitals Cleveland Medical Center, Cleveland, OH USA; 8 Department of Population & Quantitative Health Sciences, Case Western Reserve University, Cleveland, OH USA

**Keywords:** Cellular immunity, Biomarkers, Tuberculosis, Antibodies

## Abstract

Exposure to *Mycobacterium tuberculosis* (*Mtb*) results in heterogeneous clinical outcomes including primary progressive tuberculosis and latent *Mtb* infection (LTBI). *Mtb* infection is identified using the tuberculin skin test and interferon-γ (IFN-γ) release assay IGRA, and a positive result may prompt chemoprophylaxis to prevent progression to tuberculosis. In the present study, we report on a cohort of Ugandan individuals who were household contacts of patients with TB. These individuals were highly exposed to *Mtb* but tested negative by IFN-γ release assay and tuberculin skin test, ‘resisting’ development of classic LTBI. We show that ‘resisters’ possess IgM, class-switched IgG antibody responses and non-IFN-γ T cell responses to the *Mtb*-specific proteins ESAT6 and CFP10, immunologic evidence of exposure to *Mtb*. Compared to subjects with classic LTBI, ‘resisters’ display enhanced antibody avidity and distinct *Mtb*-specific IgG Fc profiles. These data reveal a distinctive adaptive immune profile among *Mtb*-exposed subjects, supporting an expanded definition of the host response to *Mtb* exposure, with implications for public health and the design of clinical trials.

## Abstract (web summary)

New immune biomarkers of exposure to tuberculosis may require a rethink of evidence of *Mycobacterium tuberculosis* infection and control.

## Main

*Mycobacterium tuberculosis* (*Mtb*) is the leading infectious cause of death worldwide^1^. Exposure to *Mtb* leads to a spectrum of outcomes, including primary progressive disease and latent *Mtb* infection (LTBI). A diagnosis of LTBI is based on evidence of immune sensitization to *Mtb* antigens and the absence of clinical symptoms of tuberculosis (TB) or direct microbiologic evidence of disease^2^. The clinical standards that establish evidence of *Mtb* exposure and infection include the tuberculin skin test (TST) and interferon-γ (IFN-γ) release assay (IGRA). TST measures a delayed-type hypersensitivity reaction to purified protein derivative (PPD) from *Mtb*^2,3^. The IGRA was developed to distinguish between bacille Calmette–Guèrin (BCG) vaccination and *Mtb* infection via the ex vivo measurement of T cell-produced IFN-γ to peptides from the *Mtb* proteins ESAT6 and CFP10 (ref. ^2,3^).

A subset of healthy, immunocompetent individuals remain TST and IGRA negative despite persistent, high levels of exposure to *Mtb*^4,5^. Highly *Mtb*-exposed but persistently TST and IGRA negative individuals have been described among healthcare workers^6,7^, household contacts of patients with TB^8–11^ and gold miners living and working in close quarters with individuals with active disease^12,13^. These individuals have been dubbed ‘resisters’ related to their persistent ability to remain TST and IGRA negative^4,5^. Initial studies seeking to define why these individuals continue to remain TST/IGRA negative despite high Mtb exposure identified two associated chromosomal loci^14^. In addition, differences in transcriptional responses to *Mtb* infection were found in blood monocytes from these individuals compared with individuals who develop a positive TST after similar mycobacterial exposure^15^. These innate signatures point to potentially unique, first-response immunity to *Mtb*. From the adaptive immune perspective, the persistent lack of TST and IGRA reactivity has been interpreted as suggesting that these individuals are uninfected despite long-term close-contact exposure. Another explanation could, however, be that the ‘resister’ phenotype reflects an alternative immune response to *Mtb* exposure and/or infection.

In the present study, we sought to explore the immunologic basis of persistent TST and IGRA negativity. We leveraged a longitudinal cohort study in Uganda^11^ to identify ‘resisters’, a population of household contacts who were highly exposed to *Mtb* yet remained persistently IGRA and TST negative over an average of 9.5 years of follow-up for each individual. ‘Resisters’ did not possess a natural or generic anti-pathogen-specific antibody profile that could account for a unique ability to handle *Mtb*. Instead, they possessed IgM and also class-switched IgG and IgA to several *Mtb* antigens, suggestive of extended exposure and T cell help. Moreover, T cell responses to *Mtb* antigens were detected, marked by antigen-specific upregulation of CD40L/CD154, a co-stimulatory molecule facilitating antigen-specific B cell maturation. *Mtb*-specific humoral immunity among ‘resisters’ exhibited enhanced avidity, skewing toward the IgG1 subclass selection, and distinct IgG Fc-glycosylation profiles compared with matched household contacts who converted their TST and IGRA, indicative of classic LTBI. These data reveal a durable and unique adaptive immune profile after *Mtb* exposure not captured within the current clinical spectrum of disease and expand the range of TB responses, informing future immune correlate-guided interventions.

## Results

### A subset of highly *Mtb*-exposed adults ‘resist’ developing traditional TST- and IGRA-positive LTBI

*Mtb* infection is acquired primarily via aerosol transmission through close contact with an individual with pulmonary TB. Nevertheless, in household contact studies not all individuals with high levels of *Mtb* exposure become infected, as measured by TST and IGRA^9^. In the present study we aimed to more fully characterize the immune responses *to Mtb* in these individuals to determine whether they are truly non-reactive to *Mtb* or, alternatively, have non-canonical responses after exposure.

A longitudinal cohort in Uganda was established to identify and follow individuals prospectively between 2002 and 2012 who remained persistently TST negative (PTST–) and IGRA negative despite high exposure to *Mtb* in household contacts of pulmonary TB^9^. High exposure in this area^1^ and within the households was determined using an epidemiologic risk score^16^ built on proximity and clinical characteristics of the index case. This score was used to ensure all subjects were highly and equally exposed across subject groups. Among 2,585 household contacts of 872 individuals with pulmonary TB, 173 (7.3%) were diagnosed with active TB and 1,954 (82.1%) with LTBI by TST on initial enrollment. Of these household contacts, 198 (8.3%) were PTST– upon repeated testing over 2 years of follow-up despite equivalent epidemiologic risk profiles to contacts diagnosed with LTBI.

**Extended Data Fig. 1:**
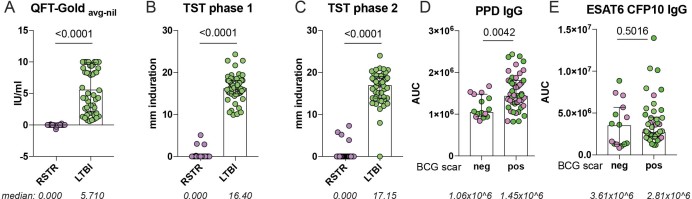
Stratification of ‘resisters’ and LTBI controls by IGRA, TST and BCG scar. **a**–**c**, IGRA results (**a**) are shown by the average of three sequential QFT-Gold readings minus the background (nil) stratified across RSTR (*n* = 40) or LTBI (*n* = 39). TST results, stratified across RSTR or LTBI, are reported in mM of induration measured in phase 1 (**b**) and phase 2 (**c**) of the clinical trial. Medians are depicted by lines with interquartile ranges. **d**,**e**, Plasma levels of IgG reactive to PPD (**d**) and ESAT6 and CFP10 (**e**) were quantified in RSTR (*n* = 40) and LTBI (*n* = 39) individuals using a customized multiplex Luminex in serial dilutions. AUCs were determined from MFIs generated with each dilution and plotted for each individual. Data points are stratified by BCG scar status. Statistical significance was calculated by Mann–Whitney U test and two-tailed *P* values are indicated.

Although most conversions in the cohort occurred rapidly, we aimed to determine the durability, stability and long-term outcomes across the 144 contacts who remained PTST– and 303 contacts with traditional LTBI who had equivalent baseline clinical and epidemiologic risk scores. Specifically, a re-tracing study was performed in 2014–2017 at an average of 9.5 years after initial *Mtb* exposure^9,16^. Three sequential IGRAs, measured by QuantiFERON-TB Gold, were performed on blood samples and one additional TST was performed at the end of the re-tracing study. Of the original TST population, 82.7% remained PTST– and IGRA negative. Although there were small groups of individuals with conversions and reversions of TST and IGRAs, only human immunodeficiency virus (HIV)-negative subjects who remained concordantly negative for all tests (*n* = 82) were defined as ‘resisters’ and used in this analysis (see Supplementary Table 1)^11^. By extension, HIV-negative control subjects with LTBI were defined by consistently positive results at all time points by both IGRA (Extended Data Fig. 1a) and TST (Extended Data Fig. 1b,c), with no evidence of clinical disease. To balance for confounding factors, a representative subset (see Supplementary Table 2) of ‘resisters’ and LTBI controls were matched by age (≥15 years), gender and epidemiologic risk score (see Supplementary Table 1), representing, to our knowledge, the longest followed cohort of ‘resisters’. To assess immune responses, peripheral blood samples obtained during the re-tracing study, reflecting cumulative experience of *Mtb* exposure during the initial TB household contact study and subsequent years in a TB-endemic urban environment, were used for further analysis.

### Limited evidence for differential natural and non-*Mtb* antibodies among ‘resisters’

**Fig. 1:**
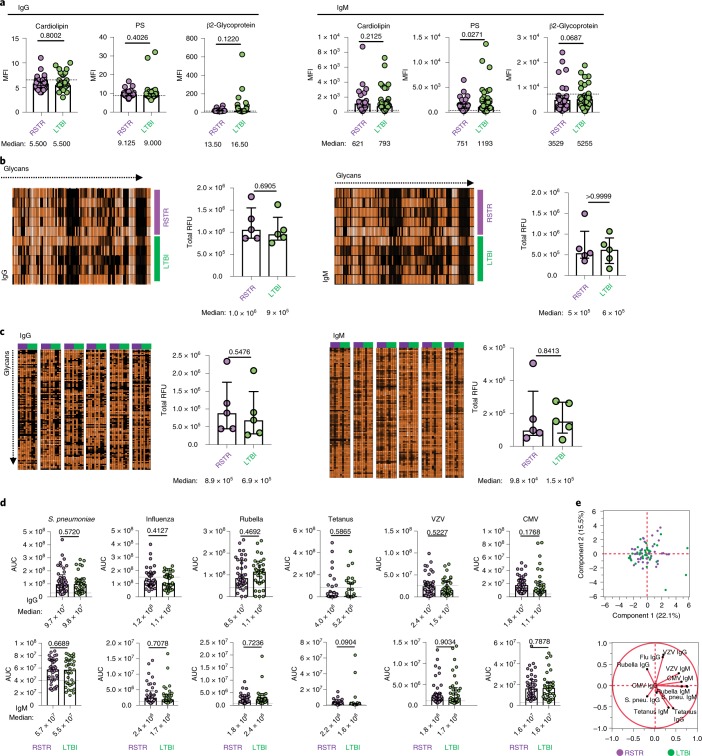
Comparable antibody reactivity to natural, glycan and common pathogens in ‘resisters’ and LTBI controls. **a**, Plasma levels of IgG and IgM reactive to the common natural antigens cardiolipin, phosphatidylserine (PS) and β_2_-glycoprotein were profiled across ‘resisters’ (RSTR) (*n* = 40) and LTBI individuals (*n* = 39), represented by MFI using a customized Luminex with medians and interquartile ranges depicted for each group. **b**,**c**, Plasma IgG and IgM reactivity to 700 glycans in age- and sex-matched ‘resisters’ (*n* = 5) and LTBI individuals (*n* = 5) were determined on the NCFGv1 glycan microarray (**b**) and the CFG mammalian-type glycan microarray CFGv5 (**c**). Total fluorescence intensities depicted in heatmaps were determined per individual (rows in **b** and columns in **c**) and plotted in dot plots as relative fluorescence units (RFU), with medians and interquartile ranges depicted for each group. **d**, Plasma levels of IgG and IgM reactive to *Streptococcus pneumoniae* capsular polysaccharides (S. pneu.), a mixture of influenza HA, rubella virus, tetanus toxoid, VZV and CMV pp65 in ‘resisters’ (*n* = 40) and LTBI individuals (*n* = 39), were determined using customized multiplex Luminex. AUCs were determined from MFIs generated by three dilutions and plotted for each individual with medians and interquartile ranges depicted for each group. For **a**–**d**, statistical significance was calculated using the Mann–Whitney U test, and two-tailed *P* values are indicated. Dotted lines represent the median level detected in HIV-negative, healthy North American volunteers. **e**, Principal component analysis using the IgG and IgM data generated in **d** demonstrates overlapping dot plots of microbial-reactive antibody profiles in ‘resisters’ (*n* = 40) and LTBI individuals (*n* = 39). The loadings plot and PLSDA plot are mirror images. Thus, the geographic location of the 12 antibody features on the loadings plot reflects the subject’s group in which a particular feature is enriched.

Given the emerging appreciation of a role for antibodies in TB^17–21^, we first hypothesized that ‘resisters’ may have high levels of natural antibodies that might provide protection from infection. Plasma collected at the time of enrollment into the re-tracing study was used to profile natural IgG and IgM levels against classic natural antibody targets, such as cardiolipin, phosphatidylserine and β_2_-glycoprotein. However, no differences were observed in antibody titers between 39 ‘resisters’ and 40 matched LTBI controls (Fig. 1a).

Given the complexity of glycosylated lipids and proteins in *Mtb*, we next hypothesized that ‘resisters’ might have a propensity to target carbohydrate antigens more readily than control subjects. The reactivity of antibodies to more than 600 distinct glycans was assessed using two arrays: the National Center for Functional Genomics (nCFG) array, composed of 100 mammalian, microbial and plant-derived glycans, and the larger Consortium for Functional Glycomics (CFG) array, composed of 609 mammalian, microbial, milk and arthropod glycans^22,23^. No differences were observed in overall glycan reactivity between ‘resisters’ and LTBI controls across either IgG or IgM (Fig. 1b,c).

We also hypothesized that ‘resisters’ may respond to vaccination or infection differentially, mounting a unique response to respiratory pathogens including *Mtb*. Thus, we profiled the humoral immune response to a number of pathogen and vaccine antigens. No statistically significant differences were observed in the humoral responses to antigens from respiratory pathogens (*S. pneumoniae* and influenza) (Fig. 1d) and non-respiratory pathogens (varicella-zoster virus (VZV), cytomegalovirus (CMV), rubella and tetanus), across IgG and IgM. Multivariate analysis of the antibody reactivity across the two groups showed complete overlap (Fig. 1e), providing no evidence of differences in general immunologic humoral reactivity between ‘resisters’ and LTBI controls.

### Robust *Mtb*-specific humoral immune responses among ‘resisters’

**Fig. 2:**
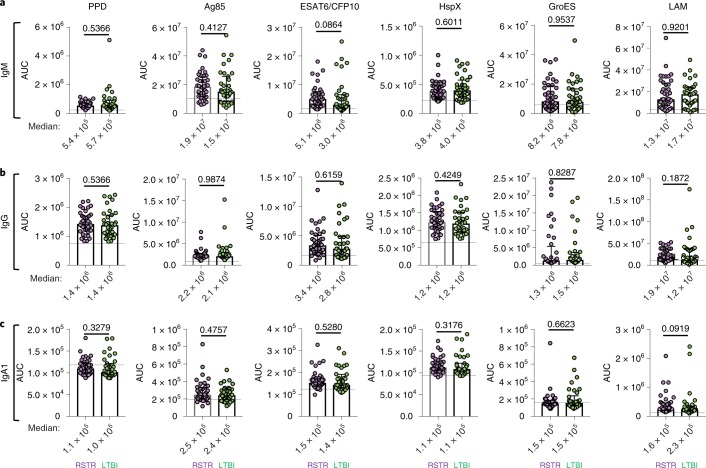
Detectable *Mtb*-specific humoral immunity in ‘resisters’. **a**–**c**, Plasma levels of IgM (**a**), IgG (**b**) and IgA1 (**c**) reactive to PPD, Ag85A, ESAT6 and CFP10, α-crystalline (HspX), GroES and LAM were quantified in ‘resisters’ (*n* = 40) and LTBI individuals (*n* = 39) with AUCs determined from MFIs generated using a customized Luminex assay, generated with three dilutions and plotted for each individual with medians and interquartile ranges depicted for each group. The statistical significance was calculated using the Mann–Whitney U test, and two-tailed *P* values are indicated. Dotted lines represent the median level detected in HIV-negative, healthy North American volunteers.

We then assessed humoral immune responses to *Mtb*-associated protein and glycan antigens. As a result of the negative TST and IGRA results, we hypothesized that these responses would be absent among ‘resisters’ but detectable among LTBI control subjects. Surprisingly, IgM (Fig. 2a), IgG (Fig. 2b) and IgA (Fig. 2c) reactivity was observed to all *Mtb* antigens tested (purified protein derivative (PPD), antigen 85 (Ag85), ESAT6/CFP10, the latency-associated protein HspX, the chaperone protein GroES and *Mtb* cell-wall lipoarabinomannan or LAM) in both ‘resisters’ and matched LTBI individuals. Importantly, ‘resisters’ had antibody responses of all isotypes against ESAT6 and CFP10, which are used to distinguish *Mtb* exposure from BCG vaccination in the IGRA. In both ‘resisters’ and matched LTBI controls, antibody responses were higher than those detected in healthy individuals from a non-endemic region, which serve as a technical benchmark for the assay. Thus, although ‘resisters’ are defined by the lack of IFN-γ-dependent T cell immunity to ESAT6 and CFP10 by IGRA, these individuals possess persistent antibody responses to these same antigens, providing immunologic evidence of exposure to *Mtb*. Furthermore, these antibody responses have undergone class switching, arguing for concomitant T cell responses which may be distinct from those identified by IGRA. These data are consistent with the anti-*Mtb* humoral responses reported in a study of Chinese healthcare workers who were highly exposed but TST and IGRA negative^24^.

### ‘Resisters’ possess *Mtb*-specific IFN-γ-negative T cell responses

**Fig. 3:**
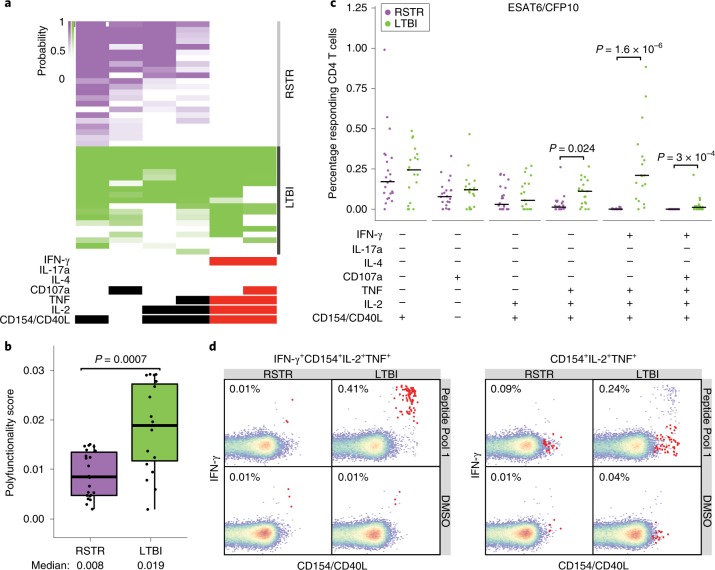
‘Resisters’ display IFN-γ-independent T cell responses to *Mtb*-specific protein antigens. **a**, ICS data generated using *Mtb-*specific proteins ESAT6 and CFP10 (Peptide Pool 1) were analyzed using COMPASS, and the results from six functionally relevant T cell subsets are displayed as a heatmap of the probability of detecting a response above background. Subsets containing IFN-γ are noted in red. Rows represent study subjects and the columns represent CD4 T cell functional subsets. **b**, Subject-specific COMPASS results were summarized for 41 individuals using the polyfunctionality score, which weights T cell subsets that include more than one function. Medians and interquartile ranges are depicted. The statistical significance was calculated using the Mann–Whitney U test, and the two-tailed *P* value is indicated. **c**, The absolute magnitude of responding CD4 T cells after background correction is displayed for each of the functional subsets identified by COMPASS. Individual data points for *n* = 41 (22 ‘resisters’, 19 LTBI controls) are shown with bars indicating medians. To facilitate visualization, we have not displayed a single LTBI outlier with a value of 3.12%. Statistical testing was performed using the Mann–Whitney U test, with correction for multiple hypothesis testing using Bonferroni’s method, and two-tailed *P* values are depicted. **d**, Representative flow cytometry plots from a ‘resister’ and a control subject examining an IFN-γ-containing or an IFN-γ-lacking T cell subset in response to stimulation with DMSO or Peptide Pool 1, with each experiment performed once. Frequencies of the relevant T cells are shown and indicated as red dots in the typical two-dimensional layout.

**Extended Data Fig. 2:**
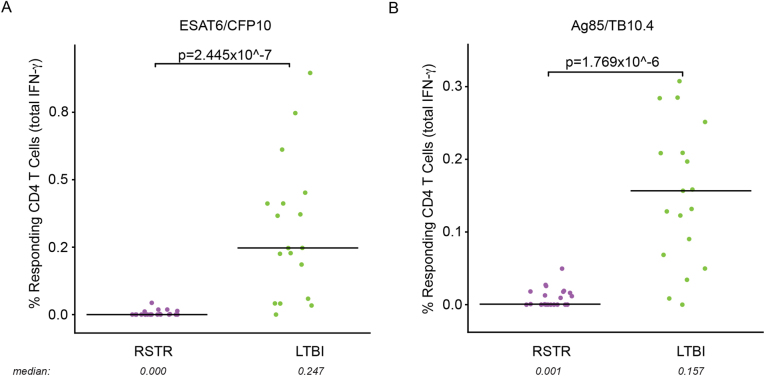
Analysis of total IFN-γ production by CD4 T cells after stimulation with peptide pools. **a**,**b**, Aggregate IFN-γ-positive events, irrespective of the production of other cytokines, in response to stimulation with ESAT6/CFP10 (**a**) or Ag85/TB10.4 (**b**) are plotted. Individual data points for *n* = 41 are displayed after background correction, stratified by cohort assignment (RSTR or LTBI) with medians represented by lines. To facilitate visualization, we have not displayed a single LTBI outlier with value 3.41 and 4.25% in **a** and **b**, respectively. Statistical testing was performed on all the data points using Mann–Whitney U test and unadjusted two-tailed *P* values are shown.

Given our emerging appreciation for the complexity of T cell functions in different infections^25^, we tested for *Mtb*-specific, IFN-γ-independent, CD4^+^ T cell immunity. Peripheral blood mononuclear cells (PBMCs) from 25 ‘resisters’ and 21 LTBI control subjects, matched for age, sex and epidemiologic risk score (see Supplementary Table 1), were analyzed. PBMCs were stimulated with overlapping peptide pools targeting ESAT6/CFP10, and assessed for seven T cell functions using a previously validated intracellular cytokine staining (ICS) assay, capturing distinct T cell functional subsets producing interleukin (IL)-2, IL-4, IL-17a, IFN-γ, tumor necrosis factor (TNF) and CD107a, and expressing CD40L/CD154 (ref. ^26,27^.). A theoretical 128 possible combinations of T cell functions were captured, 64 of which included IFN-γ. To ensure the detection of IFN-γ-negative T cells that might be present at extremely low frequencies in this high-dimensional analysis, combinatorial polyfunctionality analysis of antigen-specific T cell subsets (COMPASS) was employed^28,29^. As expected, we detected polyfunctional CD4 T cell responses to ESAT6/CFP10, which included IFN-γ among almost all LTBI subjects. Two subjects with LTBI did not show IFN-γ responses. This was attributed to decreased sensitivity from a short incubation time (6 h compared with overnight in standard IGRA testing). In contrast, no IFN-γ responses were observed among ‘resisters’, accounting for the reduced polyfunctionality score compared with LTBI subjects (Fig. 3a,b). The absolute magnitude of responding CD4 T cells showed the same profile observed in the COMPASS analysis (Fig. 3c and Extended Data Fig. 2). However, ‘resisters’ did exhibit detectable ESAT6/CFP10-specific CD4^+^ T cell responses characterized by the absence of IFN-γ and the presence of TNF^+^IL-2^+^CD40L/CD154^+^, IL-2^+^CD40L/CD154^+^, CD40L/CD154 alone or CD107a alone (Fig. 3a,c). The absolute magnitudes of IFN-γ-independent T cell responses among ‘resisters’ were less than those among LTBI subjects but consistently above the background level (Fig. 3c,d). IFN-γ-independent T cell responses were also detected among household contacts who developed LTBI but were not universally present in TB-endemic populations, as we have shown previously^28^. Thus, even though ‘resisters’ do not meet clinical diagnostic criteria for *Mtb* infection or disease, they possess IFN-γ-independent CD4 T cell responses to ESAT6/CFP10, consistent with the presence of class-switched humoral immunity to *Mtb* antigens.

### ‘Resisters’ display reduced CD4-mediated IFN-γ responses across *Mtb* antigens

**Fig. 4:**
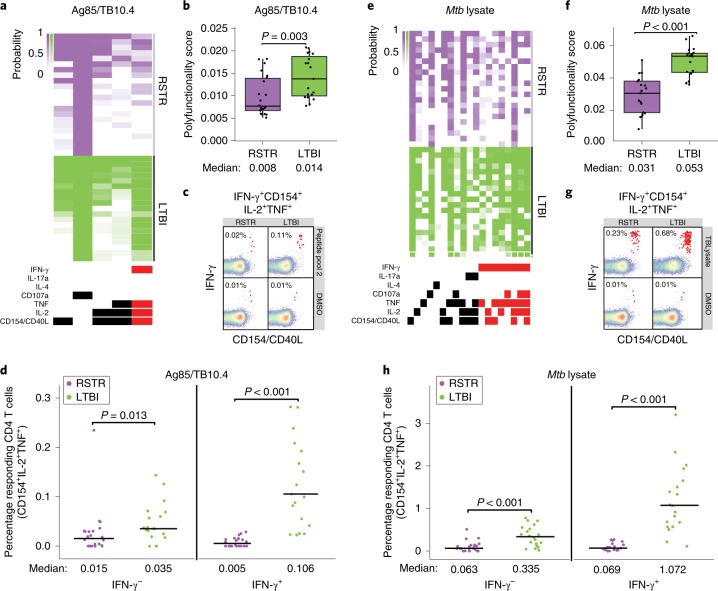
‘Resisters’ have decreased T cell responses to common mycobacterial antigens. **a**, COMPASS analysis identified five functionally relevant CD4 T cell subset responses to Peptide Pool 2 (Ag85A, Ag85B and TB10.4), which are summarized in the heatmap. Rows represent study subjects and columns CD4 T cell functional subsets. The depth of shading within the heatmap represents the probability of detecting a response above background. IFN-γ-containing subsets are noted in red. **b**, Subject-specific COMPASS results in response to stimulation with Peptide Pool 2 were summarized using the polyfunctionality score, which weights T cell subsets that include more than one function. The total number of subjects analyzed was 41. Boxplots show median and interquartile ranges. The statistical significance was calculated using the Mann–Whitney U test, and the two-tailed *P* value is indicated. **c**, Representative flow cytometry plots from a ‘resister’ and an LTBI subject show frequencies of IFN-γ^+^CD40L/CD154^+^IL-2^+^TNF^+^ T cells (red dots) in response to stimulation with Peptide Pool 2 or DMSO, with each experiment performed once. **d**, The absolute magnitude of Ag85/TB10.4-specific polyfunctional CD40L/CD154+ IL-2+ TNF+ CD4 T cells after background correction is displayed stratified by the expression of IFN-γ. These functional subsets represent the two right columns of the COMPASS plot in **a**. To facilitate visualization, we have not displayed a single LTBI outlier with the value of 3.41%. The total number of subjects analyzed was 41. Lines identify medians. The statistical significance was calculated using the Mann–Whitney U test, and two-tailed *P* values are indicated. **e**, COMPASS analysis identified 19 functionally relevant CD4 T cell subset responses to *Mtb* lysate, which are summarized in the heatmap. IFN-γ-containing subsets are noted in red. **f**, Polyfunctionality scores for the 41 subjects in response to *Mtb* lysate stimulation are shown, with boxplots representing median and interquartile ranges. The statistical significance was calculated using the Mann–Whitney U test, and the two-tailed *P* value is shown. **g**, Representative flow cytometry plots from a ‘resister’ and TST/IGRA-positive subject showing frequencies of IFN-γ^+^CD40L^+^IL-2^+^TNF^+^ T cells (red dots) in response to stimulation with *Mtb* lysate or DMSO are shown with each experiment performed once. **h**, The absolute magnitude of *Mtb* lysate-specific polyfunctional CD154^+^IL-2^+^TNF^+^ CD4 T cells after background correction is displayed, stratified by the expression of IFN-γ for 40 subjects, with lines representing medians. These functional subsets represent the 9th and 17th columns of the COMPASS plot in **e**. To facilitate visualization, we have not displayed a single LTBI outlier with a value of 12.61%. The statistical significance was calculated using the Mann–Whitney U test, and unadjusted two-tailed *P* values are shown.

**Extended Data Fig. 3:**
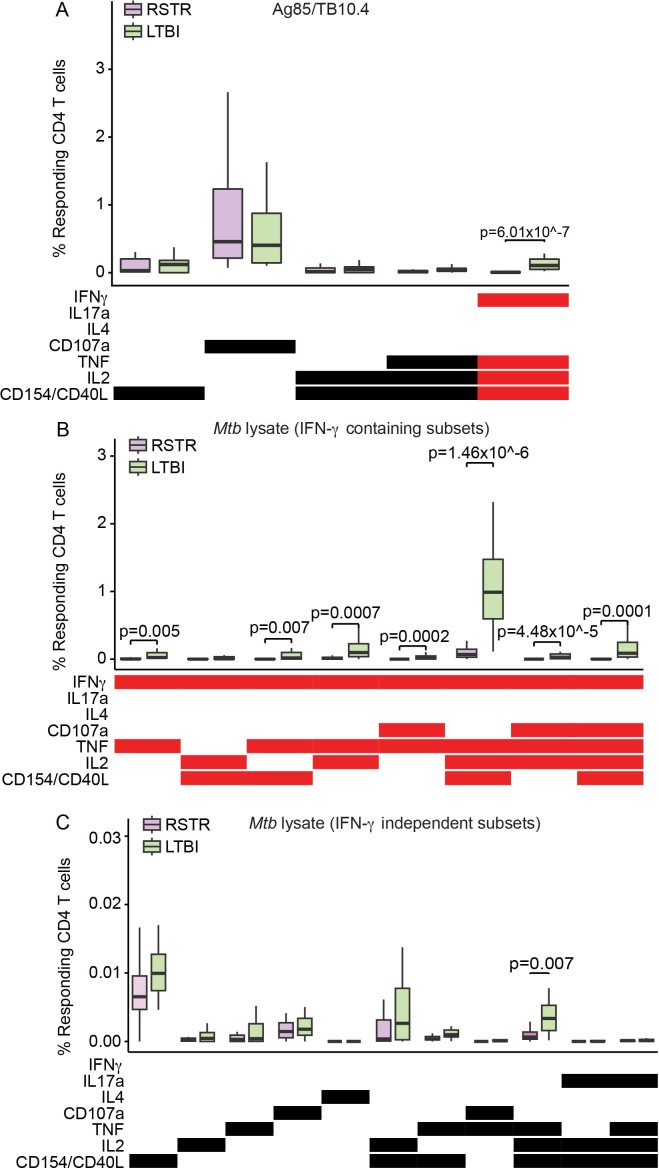
Frequencies of CD4 T cell functional subsets identified by COMPASS after stimulation with Ag85/TB10.4 and *Mtb* lysate. **a**, Absolute magnitude after background correction of CD4 T cell functional subsets identified by COMPASS in Fig. 4a upon stimulation with Ag85/TB10.4 are shown for RSTRs (*n* = 22) and LTBI (*n* = 19). **b**,**c**, For CD4 T cell subsets identified by COMPASS after *Mtb* lysate stimulation in Fig. 4e, absolute magnitude after background correction is shown for IFN-γ-containing (**b**) or IFN-γ-independent (**c**) T cell subsets. Boxplots show median, 25th and 75th percentile of the distribution and whiskers depict the range without outliers. IFN-γ-containing subsets are noted in red. Statistical testing was performed on all the data points using Mann–Whitney U test with correction for multiple hypothesis testing using the Bonferroni method and two-tailed *P* values are shown.

**Extended Data Fig. 4:**
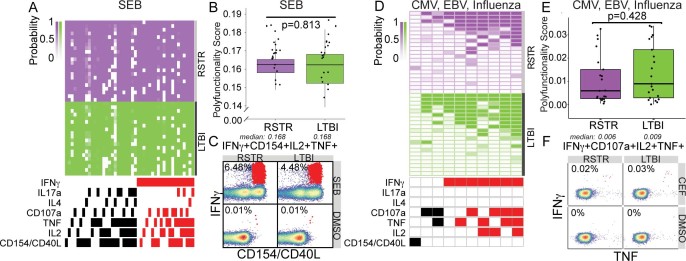
‘Resisters’ are not globally deficient in IFN-γ production by T cells. **a**, The COMPASS heatmap shows 49 informative CD4 T cell subset responses to staphylococcus enterotoxin B (SEB) for *n* = 42 individuals. Rows represent study subjects and columns represent CD4 T cell functional subsets. The depth of shading within the heatmap represents the probability of detecting a response to a given subset in a given subject above background. IFN-γ-containing subsets are noted in red. **b**, Subject-specific COMPASS results in response to SEB stimulation were summarized using a polyfunctionality score, which weights T cell subsets that include more than one function. Boxplots show median and interquartile range. Statistical significance was calculated by Mann–Whitney U test and the two-tailed *P* value is shown. **c**, Representative flow cytometry plots from a ‘resister’ and LTBI subject show frequencies of CD154+IFN-γ+IL-2+TNF+ T cells (red dots) in response to stimulation with SEB or DMSO, with each experiment performed once. **d**, The COMPASS heatmap shows 10 informative CD8 T cell subset responses to CMV, Epstein Barr virus (EBV) and influenza (CEF) combined peptide pool. IFN-γ containing subsets are noted in red. **e**, Polyfunctionality scores are depicted for *n* = 21 RSTRs and *n* = 21 LTBI subjects with boxplots show median and interquartile ranges. Statistical significance was calculated by Mann–Whitney U test and the two-tailed *P* value is shown. **f**, Representative flow cytometry plots from a ‘resister’ and LTBI subject display nearly equivalent frequencies of CD107a+IFN-γ+IL-2+TNF+ T cells (red dots) in response to stimulation with CEF or DMSO, with each experiment performed once.

Next, we sought to more broadly characterize CD4 T cell immunity to mycobacteria among ‘resisters’. PBMCs were stimulated with overlapping peptide pools targeting Ag85A, Ag85B, TB10.4 and *Mtb* lysate. These antigens are expressed across a larger array of mycobacterial species compared with the restricted expression of ESAT6 and CFP10, reflecting exposure to non-tuberculous mycobacteria or BCG vaccination. COMPASS analysis following CD4 T cell stimulation with Ag85/TB10.4 overlapping peptides (Fig. 4a,b) showed detectable but overall reduced absolute magnitudes of both IFN-γ-containing and IFN-γ-independent T cell populations (Fig. 4c,d and Extended Data Figs. 2 and 3) in ‘resisters’ compared with LTBI individuals. Results obtained after stimulation with *Mtb* lysate were also consistent with this pattern (Fig. 4e,h, Extended data Fig. 3). As these data were similar to stimulation with ESAT6/CFP10, the possibility that ‘resisters’ may be globally deficient in IFN-γ production by T cells was raised. However, stimulation with staphylococcal enterotoxin B (SEB) induced comparable levels of IFN-γ production among CD4 T cells in both ‘resisters’ and LTBI subjects (Extended Data Fig. 4a–c). Also, stimulation with peptides specific for CMV, Epstein–Barr virus and influenza demonstrated comparable levels of IFN-γ production from CD8 T cells in both groups (Extended Data Fig. 4d–f). Thus, overall, ‘resisters’ generate reduced IFN-γ-producing CD4 T cells to mycobacterial antigens in the absence of any evidence of compromised overall IFN-γ/T-helper 1 (Th1) immunity.

### ‘Resisters’ generate qualitatively distinct, *Mtb*-specific, humoral immune responses

**Fig. 5:**
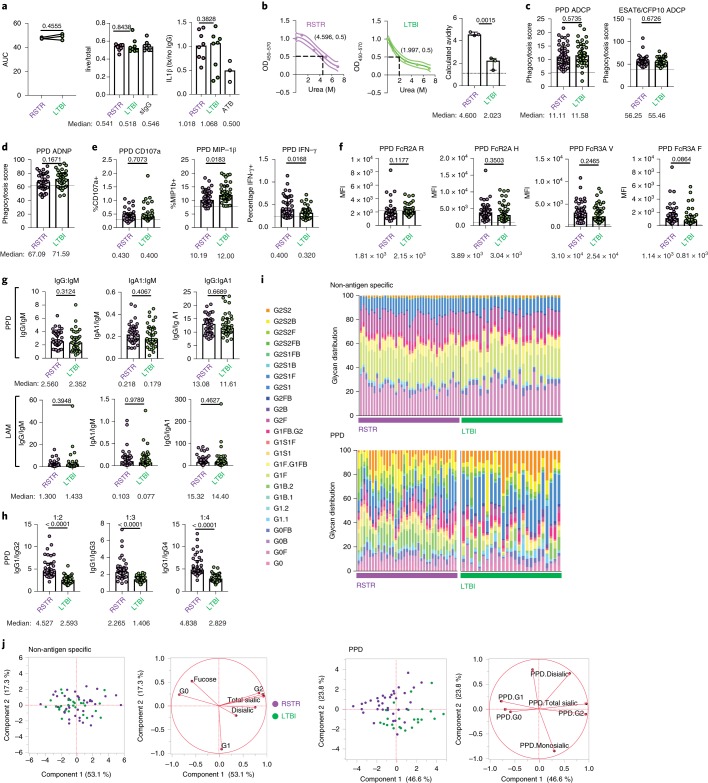
Qualitatively distinct PPD-specific antibody responses in ‘resisters’ compared with LTBI individuals. **a**, Graphs depict the AUCs calculated from the ratio of live to total intracellular bacterial burden in primary human monocyte-derived macrophages after treatment with purified IgG at 0.1 mg ml^–1^, 0.01 mg ml^–1^ and 0.001 mg ml^–1^ (left) from ‘resisters’ (*n* = 40) and TST/IGRA-positive LTBI controls (*n* = 39). Extension of analysis to additional donors was performed at a single concentration of purified IgG of 0.1 mg ml^–1^ due to sample availability (middle). Levels of secreted IL-1β from supernatants were measured by ELISA and are shown relative to no antibody treatment. Purified IgG from individuals in this study, with culture-confirmed pulmonary TB (ATB), is shown as a benchmark. Each line represents one healthy macrophage donor individual. For dot plots, lines are medians. The statistical significance was calculated using Wilcoxon’s matched-pairs signed rank, and two-tailed *P* values are shown. **b**, The calculated avidity against PPD from pooled plasma from ‘resisters’ (*n* = 40), TST/IGRA-positive LTBI controls (*n* = 39) and healthy, HIV-uninfected North Americans (*n* = 10) are shown, with lines representing the fitted curves and dotted lines the 95% confidence intervals. Each plasma group was tested in triplicate, with associated calculated avidity represented in the dot plot. The statistical significance was calculated using the Student’s *t*-test, and a two-tailed *P* value is indicated. OD, optical density or absorbance. **c**–**e**, Plasma from ‘resisters’ (*n* = 40) and LTBI controls (*n* = 39) was assayed for the ability to mediate: PPD and ESAT6/CFP10-specific, antibody-dependent, monocyte-mediated cellular phagocytosis (**c**); PPD-specific, antibody-dependent neutrophil phagocytosis (**d**); and PPD-specific, NK cell activation by CD107a expression, macrophage inflammatory protein-1β and IFN-γ production (**e**). Data are representative of experiments performed in duplicate over three dilutions. Assays utilizing primary human neutrophils (**d**) and NK cells (**e**) were additionally performed utilizing three independent, healthy, HIV-negative donors. **f**, Affinity for FcγR2A(R), FcγR2A(H), FcγR3A(V) and FcγR3A(F) were determined using customized Luminex to PPD in ‘resisters’ (*n* = 40) and LTBI individuals (*n* = 39), using plasma diluted at 1:100. MFI is shown on the graph. The statistical significance was calculated using the Mann–Whitney U test, and *P* values are indicated. Dotted lines represent the median level detected in HIV-negative, healthy North American volunteers. **g**, Ratios of plasma levels of IgM, IgG and IgA1 reactive to PPD and LAM in ‘resisters’ (*n* = 40) and TST/IGRA-positive LTBI controls (*n* = 39) are depicted with medians and interquartile ranges. The statistical significance was calculated using the Mann–Whitney U test, and two-tailed *P* values are indicated. **h**, Ratios of plasma levels of IgG1, IgG2, IgG3 and IgG4 reactive to PPD in ‘resisters’ (*n* = 40) and TST/IGRA-positive LTBI individuals (*n* = 39) were measured by customized multiplex Luminex in serial dilutions. AUCs are depicted with medians and interquartile ranges. The statistical significance was calculated using the Mann–Whitney U test, and two-tailed *P* values are indicated. **i**, The relative distribution of glycoform substructures isolated from non-antigen-specific and PPD-specific IgG are depicted, with each column representing each individual. **j**, Principal component analysis demonstrates the overlapping profiles of ‘resisters’ (*n* = 40) and TST/IGRA-positive LTBI individuals (*n* = 39) in the dominant total glycans isolated from non-antigen-specific IgG compared with partially separating profiles from PPD-specific IgG. ADCP, antibody-dependent cellular phagocytosis; ADNP, antibody-dependent neutrophil phagocytosis; MIP, macrophage inflammatory protein.

Given the distinct Th profile observed among ‘resisters’, we next probed for potential differences in the humoral immune profiles in ‘resisters’ compared with LTBI subjects. IgG from individuals with LTBI and active *Mtb* disease mediates differential intracellular bacterial restriction coupled with *Mtb*–phagolysosomal co-localization and inflammasome activation in the absence of differential opsinophagocytosis^17^. As such, we interrogated the antimicrobial potential of IgG from ‘resisters’ in an in vitro macrophage model of *Mtb* infection. No statistically significant differences were observed between the effects of ‘resister’ and LTBI IgG on macrophage intracellular bacterial burden (Fig. 5a), pointing to equivalent antimicrobial function across both groups. Similarly, IL-1β release, a marker of inflammasome activation previously linked to antimicrobial antibody *Mtb* restriction^30^, was equivalent in the presence of ‘resister’ and LTBI IgGs, both of which trended to higher levels than those induced by IgG from individuals with active pulmonary TB^17^ (Fig. 5a). These data suggest equivalent restrictive activity across ‘resisters’ and LTBI controls, previously observed to diverge from active *Mtb* disease, pointing to the generation of antimicrobial humoral immune responses in ‘resisters’ in the absence of classic Th1 immunity.

For better identification of antibody features that may diverge in ‘resisters’, the strength of binding to the complex array of proteins and peptides represented by PPD was interrogated. Notably higher PPD-specific IgG avidity was observed among the polyclonal responses from ‘resisters’ compared with LTBI control subjects (Fig. 5b). Given the bivalent nature of IgG, these data suggest that ‘resisters’ possess PPD-specific antibodies that may be more affinity matured than those found in LTBI controls.

To further probe whether changes in the Fab were accompanied by changes in the Fc of *Mtb*-specific antibodies, we compared the Fc-effector functions and isotype distribution between ‘resisters’ and LTBI controls. Similar PPD-specific, Fc-effector functional profiles were observed between ‘resisters’ and LTBI controls in monocyte phagocytosis, neutrophil phagocytosis and natural killer (NK) cell degranulation, but higher levels of PPD-specific, NK cell IFN-γ-inducing antibodies were detected in ‘resisters’ (Fig. 5c–e). Consistent with these findings, similar binding to low-affinity Fc-receptor variants (FcγR2a and FcγR3a) was observed across the two groups, with an expected trend toward higher FcγR3a binding in ‘resisters’ (Fig. 5f). In addition, similar isotype ratios were observed across the two groups (Fig. 5g). In contrast, subclass selection differences were observed between the ‘resisters’ and the LTBI controls (Fig. 5h). Specifically, statistically significant skewing toward IgG1 was observed in subclass ratios in ‘resisters’ compared with LTBI controls (Fig. 5h). These data suggest that additional IgG subclasses evolve in individuals who develop LTBI whereas ‘resisters’ maintain a focused IgG1 response to *Mtb* antigens.

**Extended Data Fig. 5:**
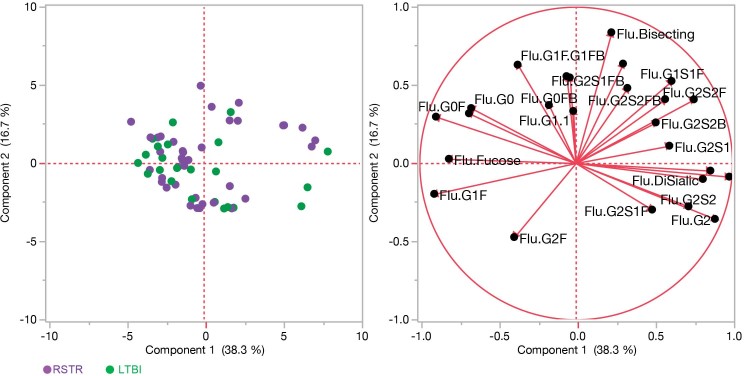
Overlapping influenza HA-specific, IgG glycan distributions between ‘resisters’ and LTBI controls. Principal component analysis demonstrates the overlapping profiles of ‘resisters’ (purple, *n* = 22) and TST/IGRA-positive LTBI (green, *n* = 19) individuals in the glycoform substructures isolated from influenza HA-specific IgG. The glycoform substructures of individuals are represented in the loadings plot (right), a mirror image of the dot plot (left), where the location of the glycoform substructures reflects the distribution of the individuals in the dot plot.

Beyond subclass differences, recent data suggest that differences in Fc glycosylation at a single conserved *N*-glycan residue (Asn^297^) can discriminate between LTBI and active *Mtb* disease^17^. Thus, Fc-glycan profiles were assessed across non-antigen-specific, bulk IgG, influenza hemagglutinin (HA)-specific IgG and PPD-specific IgG. No differences were observed in glycosylation patterns between ‘resisters’ and LTBI control subjects in bulk non-antigen-specific and influenza HA-specific antibodies (Fig. 5i and Extended data Fig. 5). In contrast, PPD-specific, Fc-glycan profiles diverged substantially across the groups, with elevated levels of singly galactosylated (G1), highly fucosylated, bisected and decreased sialylation in ‘resisters’ (Fig. 5i,j). The glycan structures that are selectively enriched in ‘resisters’ are distinct from those commonly analyzed by the monoclonal therapeutics community involved in antibody-dependent cellular phagocytosis or cytotoxicity. Thus, these Fc-glycan profiles point to potential non-canonical effector functions that may be produced in polyclonal humoral responses among ‘resisters’.

**Fig. 6:**
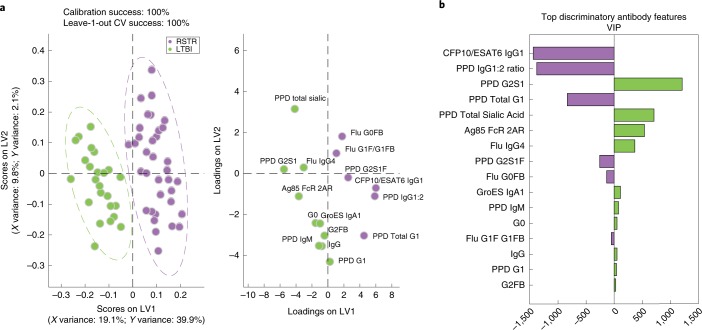
*Mtb*-specific Fc profiles segregate ‘resisters’ and LTBI controls. PLSDA was performed utilizing antigen-specific antibody levels, Fc effector functions and IgG glycosylation (total number of initial features = 216). **a**, The scores plot (left) shows the IFN-γ-negative and IFN-γ-positive profiles for each individual (dots). **b**, The loadings plot (right in **a**) shows that 16 antibody features separated each group with 100% calibration and 100% tenfold cross-validation accuracy. LV1 captures 19.1% of the *X* variance and 39.9% of the *Y* variance. Discriminatory features are depicted in a variable importance plot using the projection (VIP) scores that represent the weight of the selected variables in contributing to the overall separation between the groups (**b**) with features associated with ‘resisters’ to the left and IGRA/TST-positive LTBI individuals to the right.

**Extended Data Fig. 6:**
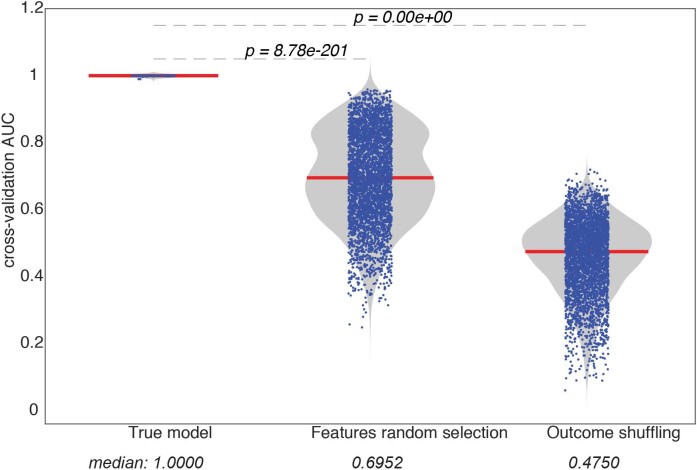
The statistical performance of the LASSO–PLSDA model. To estimate the statistical significance of the model prediction by LASSO–PLSDA, two types of permutation tests were used: (1) shuffling the outcome label and (2) selecting randomized data sets. Each permutation test was performed 1,000 times and the prediction accuracy was calculated based using a receiver operating characteristic curve (shown in the dots). The empirical null distributions for each permutation test were generated and the nominal *P* values were calculated comparing the true model to the null distributions.

To ultimately identify the minimal humoral signature that was uniquely enriched among ‘resisters’, we used a stringent multivariate model to quantitatively rank all antibody features. Collected humoral immune data were normalized and subjected to a penalty-based, least absolute shrinkage and selection operator (LASSO) to reduce highly correlated features and select the minimal number of individual antibody features capturing the overall variation among the two groups. Partial least squares discriminant analysis (PLSDA) was then used to display the data and identify the specific relationships between individual features among the groups (‘resisters’ and LTBI controls) (Fig. 6a). As few as 16 of the original 216 antibody features were required to completely separate PPD-specific antibody profiles between the groups, after taking body mass index, age and sex into account (Fig. 6b and Extended data Fig. 6). Striking separation was observed in PPD-specific Fc profiles across the two groups, resulting in 100% classification accuracy between ‘resisters’ and LTBI controls (Fig. 6a) (nominal *P* value < 0.0005 in both permutation tests, Extended data Fig. 6). The loadings plot depicts the minimal 16-feature distribution in the same multivariate space, highlighting the population of individuals in which each antibody profile feature was enriched (Fig. 6a). Consistent with analyses at an individual feature level, ESAT6/CFP10 IgG1 levels, PPD IgG1:IgG2 ratio and PPD-specific IgG-glycan features were among the top features that were enriched among ‘resisters’ (Fig. 6b). Together, these data highlight the differences in Fc profiles between the groups, marked by class-switched antibody responses with unique glycan profiles among the ‘resisters’ in the presence of a non-canonical T cell response to *Mtb* antigens.

## Discussion

Despite epidemiologic evidence of persistent exposure to *Mtb*, individuals who remain persistently TST and IGRA negative have been thought to have escaped *Mtb* infection using existing clinical tools. Using a longitudinal Ugandan study of household contacts of individuals with TB to identify the most highly exposed individuals who remained persistently TST and IGRA negative after long-term follow-up, the data presented here demonstrate that these individuals—‘resisters’—harbor humoral and non-canonical cellular immunity to *Mtb*. Specifically, we demonstrate that ‘resisters’ maintain high-titer, class-switched, affinity-matured, *Mtb*-specific antibodies with a unique Fc profile compared with matched controls. Moreover, although ‘resisters’ have the capacity to make IFN-γ in response to control antigens, they generate a non-IFN-γ-centric, *Mtb*-specific, CD4 T cell response, marked by high levels of CD40L/CD154 upregulation, which may be key to the induction of *Mtb*-specific humoral immunity. Importantly, we find that these responses to multiple *Mtb* antigens, including the antigens ESAT6 and CFP10, suggest that these responses were not generated by BCG vaccination.

In some immunocompromised states such as HIV^2,31^ and some cases of advanced disseminated TB, a persistently negative TST or IGRA is associated with loss of *Mtb* control. In the present study, ‘resisters’ are clinically well, with no signs or symptoms of clinical or subclinical immune dysfunction (Fig. 1d and Extended data Fig. 4). Moreover, TB incidence rates calculated using the last visit date of the original study and the date of re-tracing show no evidence for increased risk of progression to TB in the PTST– compared with their matched LTBI controls (see Supplementary Table 3). Based on their antibody and T cell profiles, ‘resisters’ have clearly been exposed to *Mtb* and probably have been (and may still be) infected. A larger cohort of ‘resisters’, who represent less than 10% of heavily *Mtb*-exposed individuals, is required to accurately determine whether their non-canonical immune response to *Mtb* is associated with a different risk of progression to TB compared with that of people with traditional LTBI.

Mendelian defects in IFN-γ production or signaling are associated with increased susceptibility to mycobacterial infections^32^, and studies in mice demonstrate the importance of IFN-γ in controlling *Mtb* infection^33–38^. However, several examples reveal IFN-γ-independent control of *Mtb* infection or even an antagonistic role for IFN-γ^39–41^. Moreover, in BCG-vaccinated children, low-level IFN-γ-producing T cells do not represent an immunologic correlate of risk for the development of TB^42^ and IFN-γ T cell immunity failed to predict protection against progression to *Mtb* disease after MVA85A vaccination, known to induce strong IFN-γ responses^43^. Indeed, ESAT6-specific T cells have been shown to control *Mtb* infection in the absence of IFN-γ and TNF^39^. Thus immune factors beyond IFN-γ and classic Th1 immunity may provide protection against *Mtb* disease.

The data presented here suggest that ‘resisters’ may represent an overlooked clinical outcome after *Mtb* exposure. The presence of class-switched, *Mtb*-specific immunity, in the setting of IFN-γ-independent T cells targeting ESAT6/CFP10, demonstrates that these individuals have not escaped *Mtb* exposure^4,5^ while living with an adult with pulmonary TB^16^ and residing in a TB-endemic, urban African environment^1^. Instead ‘resisters’ harbor B and T cell immunity specific for the tubercle bacillus with CD4 Th responses linked to quantitative and qualitative differences in IgG profiles to ESAT6/CFP10, LAM and PPD, compared with their matched LTBI counterparts (see Fig. 2). Persistent antibody titers equivalent to those observed in LTBI controls argue for prolonged antigenic exposure, and thus very likely *Mtb* infection. Similarly, higher-avidity IgG antibodies, observed in ‘resisters’, argue for enhanced affinity maturation and long-lasting antigen exposure (see Fig. 5). Yet, although LTBI individuals diversify their IgG subclass response, ‘resisters’ maintain an IgG1-centric state. Compared with equivalent levels of antibody-dependent phagocytosis, higher NK-cell IFN-γ secretion, linked to trending higher FcγR3a binding (see Fig. 5), recently implicated in enhanced *Mtb* control^17,44^, is observed in ‘resisters’. Finally, ‘resisters’ generate a unique, polyclonal Fc-glycan profile (see Fig. 5) marked by high levels of single galactosylated glycans. Given that antibodies bathe pulmonary tissues^45^, *Mtb*-specific antibodies may form immune complexes able to rapidly interact, regulate and direct innate and adaptive immune cell functions. Thus, further in vitro and in vivo dissection of ‘resister’-derived, antigen-specific antibody mechanism(s) of action may point to additional canonical (that is, opsinophagocytosis, complement activation and so on) and non-canonical (that is, antibody-dependent cellular toxicity, adaptive immune priming and so on) mechanisms in functional antibody responses, which track with different clinical phenotypes across the spectrum of TB.

Similar to the observed *Mtb*-specific humoral immune responses, non-IFN-γ T cell responses were detected in nearly all ‘resisters’ after ESAT6/CFP10 stimulation (see Figs. 3, 4 and Supplementary Fig. 2). These data suggest that, although ‘resisters’ do not have a defect in IFN-γ production overall, these individuals selectively develop a non-IFN-γ-producing, T cell response to *Mtb*, marked by high levels of CD40L/CD154 upregulation that is critical for T-follicular B cell help, CD107a that may drive cytotoxicity, and a variety of non-Th subset-specific cytokines that may be critical for B cell and innate activation. Whether this lack of IFN-γ reflects the generation of a distinct Th pathway (T-follicular, Th2, Th17, Th1) in response to differential exposure, major histocompatibility complex-mediated antigen processing and presentation or control of *Mtb*, or related to a blunted differentiation pathway in Th1 maturity, is unclear.

There are several models that could explain the emergence of ‘resisters’. It is possible that they become sensitized to mycobacterial antigens through BCG vaccination or exposure to environmental mycobacteria before they are exposed to *Mtb*. BCG experience appears to skew PPD immunity toward statistically significant, higher PPD-specific IgG titers (see Supplementary Fig. 1d), but, as expected, it has a limited impact on ESAT6/CFP10 profiles (Extended data Fig. 1e). Similarly, although the structure of this study was not focused on answering the question of BCG influence on *Mtb* immunity, the observation that IFN-γ production in response to cross-reactive antigens was decreased, but not completely absent, as it was with ESAT6/CFP10, suggests a potential influence of BCG on shaping immunity to shared antigens. Thus, pre-existing immunity to BCG or environmental mycobacteria could facilitate early clearance of *Mtb* after infection. Furthermore, as acquisition of IFN-γ occurs later in the differentiation of effector T cells^46^, it is possible that early clearance of antigen may arrest T cell differentiation after acquisition of IL-2 and TNF, but before IFN-γ. Future studies will be required to determine the role of vaccination, the time frame of evolution of these responses, whether these peripheral responses are reflective of site-specific immunity in the lung and how age at exposure impacts outcome.

Importantly, our data do not rule in or out persistent paucibacillary infection with *Mtb* among ‘resisters’. There is no microbiologic standard for persistent *Mtb* infection without disease. Both the IGRA and the TST, which are used currently, are informative only with respect to immune sensitization after exposure.

Collectively, the discovery of *Mtb*-specific adaptive immunity among ‘resisters’ represents a potential opportunity to explore unexpected immune correlates by expanding the spectrum of human *Mtb* infection and disease beyond that classically associated with TST and IGRA, with implications for the design of vaccine trials and public health interventions^47,48^. Future comprehensive immunologic investigative efforts of various high-exposure cohorts could provide unique insights into non-canonical immune responses^44,49^. Moreover, the discovery of validated immune correlates could significantly advance the design and testing of new therapeutics, including monoclonal antibodies and vaccines.

## Methods

### Study subjects

A total of 79 Ugandans (see Supplementary Tables 1 and 2), who were recruited from the Kawempe Community Health Study, were included in this analysis^9,51^. Index individuals with pulmonary TB were identified by culture for confirmed pulmonary TB at the Uganda National Referral Tuberculosis Treatment Center at Upper Mulago Hospital in Kampala, Uganda, between 2002 and 2012. A total of 2,585 household contacts of these index cases were enrolled and followed prospectively for up to 2 years, aimed at identifying development of LTBI based on serial TST at 0, 3, 6, 12, 18 and 24 months, or active TB based on clinical signs and symptoms of disease and culture evaluation^9^. Among all household contacts, 29.8% (*n* = 764) were TST negative at the initial visit and 10.7% of this group (*n* = 198) remained TST negative over 2 years of follow-up, that is persistently TST negative. Isoniazid preventive therapy was offered to all TST-positive contacts, and all children aged ≤ 5 years and HIV+ contacts irrespective of TST. Persistent TST– HIV– contacts aged ≥5 years were not offered isoniazid. None of the ‘resisters’ and only two LTBI individuals received chemoprophylaxis to prevent progression to TB per Ugandan national policy. From 2014 to 2017, a subset of the original cohort, specifically 162 TST-negative and matched 486 TST-positive LTBI individuals, who were 15 years of age or older at the time of this follow-up study, were eligible for re-tracing^11^; 441 (63.8%) were successfully re-contacted and willing to be re-evaluated^11^. Individuals within these two groups were matched by age, and household or epidemiological risk score^11,16,52^ (see Supplementary Tables 1 and 2). Re-traced subjects underwent a clinical evaluation including HIV testing and completed a questionnaire concerning *Mtb* exposure. HIV-uninfected individuals were selected for this study of immune responses. No isoniazid preventive therapy was provided during the re-tracing study to LTBI or ‘resister’ subjects. Among re-traced subjects, six individuals developed TB based on self-reporting (see Supplementary Table 3). These cases were used to calculate incidence rates in IGRA-positive and IGRA-negative individuals but were excluded for the immunologic analyses of this study (see Supplementary Table 2). The re-tracing study’s evaluation for *Mtb* infection status consisted of three QuantiFERON-TB Gold (QFT) assays with the first at enrollment in the re-tracing study, the next two over the next 2 years, and a TST following the last QFT. QuantiFERON-Gold-In-Tube (QFT-GIT) was used in the present study, given that this was the version of the IGRA available at the time of the re-tracing study (2014–2017), the QFT-GIT was appropriate for high-altitude settings (Kampala is at 1,200 m altitude) and, in a setting of BCG vaccination, an assay to measure *Mtb-*specific responses, was important. TST was performed using the Mantoux method (0.1 ml of 5 tuberculin units of PPD, Tubersol; Connaught Laboratories). A positive TST was defined as an induration of ≥10 mm. QFT assays were performed according to the manufacturer’s instructions and analyzed with the manufacturer’s software-generated standard curves, pass–fail criteria and definitions. In the re-tracing study 195 individuals were found to be completely concordant by two TSTs (the original from the TB contact study and one at the end of the re-tracing study) and three QFTs, and categorized as definite LTBI controls (no reversions). Eighty-two re-traced individuals were concordantly negative for the same five assays and defined as definite ‘resisters’ (Extended data Fig. 1a–c). Cryopreserved PBMCs and plasma from a subset of these definite LTBI controls and ‘resisters’ were used for the experiments in this manuscript. Sample size for antibody studies were based on our previous published study of individuals with LTBI and pulmonary TB^17^. Sample sizes for T cell studies were motivated by balancing for confounders, as well as our recently published study of South African adolescents^29^. These individuals are representative of the overall cohort (Supplementary Table 2). All study participants gave written, informed consent, approved by the institutional review boards of the participating institutions.

### IgG purification and pools

Total IgG was purified from plasma via negative selection using Melon Gel resin (Thermo Scientific) following the manufacturer’s instructions and filtered through 0.2 mM (Fisher) and 300-kDa filters (Amicon) before use. Pools of ‘resister’ and LTBI IgG were generated by mixing equivalent total IgG from each group: *n* = 40 for ‘resisters’ and *n* = 39 for TST-/IGRA-positive LTBI control individuals. The resulting pools were passed through a 300-kDa centrifugal filter (Amicon) to remove immunoglobulin complexes. IgG quantifications were determined by ELISA (eBioscience) following the manufacturer’s instructions, with each sample run in duplicate.

### Antigens

*Mtb* antigens used were: PPD (Statens Serum Institute), recombinant Ag85A and -B in a 1:1 ratio (BEI Resources, NR-14871 and NR 14870), recombinant ESAT6 (BEI Resources, NR-14868) and CFP10 in a 1:1 ratio (BEI Resources, NR-49425), HspX (provided by T. Ottenhoff), GroES (provided by T. Ottenhoff) and LAM (BEI Resources, NR-14848). A mixture of seven recombinant influenza envelope HA antigens, representative of the dominant strains in the past 10 years, was used: H1N1-A/Brisbane/59/2007, B/Florida/4/2006, B/Malaysia/2506/2004, H1N1-A/Solomon Island/3/2006, H3N2-A/Wisconsin/67/X-161/2005, H3N2-A/Brisbane/10/2007 and H1N1-A/New Caledonia/20/99 (all from Immune Technology). Whole virions (BioRad, PIP044) were used for rubella virus, purified tetanus toxoid Lp1099p (University of Massachusetts MassBiologics) for tetanus, gE(Orf68) for VZV (provided by D. Lingwood), capsular carbohydrates from the PPSV23 pneumococcal vaccine for *S. pneumoniae* and recombinant pp65 for CMV (Abcam, 43041). Cardiolipin (Sigma-Aldrich, C0563), phosphatidylserine (Sigma-Aldrich, P7769) and recombinant human apolipoprotein H/b2GP1 (R&D, P7769) were used as self-antigens. For T cell assays, *Mtb* whole cell lysate from H37Rv (BEI Resources), SEB (List Biological Laboratories, Inc.), and a cocktail of CMV, Epstein–Barr virus and influenza virus peptides (Mabtech) were used. Peptide Pool 1 consisted of ESAT6 and CFP10 (BEI Resources). Peptide Pool 2 consisted of Ag85A, Ag85B and TB10.4 (BEI Resources).

### Customized multiplex Luminex

**Extended Data Fig. 7:**
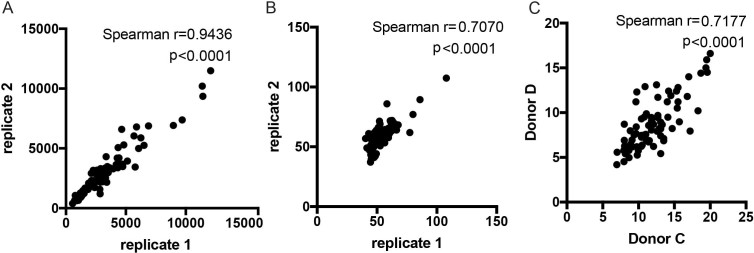
Reproducibility in antibody assays. **a**, Variability between technical replicates in customized Luminex are shown by a representative dataset of ESAT6/CFP10 specific IgG MFI readings. **b**, Variability between technical replicates in Fc-effector functional assays are shown by a representative dataset of ESAT6/CFP10 specific antibody-dependent cellular phagocytosis scores. **c**, Variability between two different donors in NK cell activation is shown by percentage Mip1b positive from Donor C and Donor D. Correlations are determined by Spearman rank with *P* values as indicated.

A Luminex isotype assay was used to quantify the relative levels of antigen-specific antibody isotypes and subclasses. Luminex Magplex carboxylated beads (Luminex) were coupled to proteins via covalent NHS-ester linkages with 1-ethyl-3-(3-dimethylaminopropyl)carbodiimide hydrochloride and NHS (Thermo Scientific) following the manufacturer’s recommendations. Glycan antigens (LAM and pneumococcal polysaccharides) were modified by 4-(4,6-dimethoxy[1,3,5]triazin-2-yl)-4-methyl-morpholinium and conjugated to Luminex Magplex carboxylated beads^53^. Lipid antigens were dissolved in ethanol and incubated with Luminex Magplex carboxylated beads^54^. Antigen-coupled beads (50 µl of a 100 microspheres µl^–1^ solution in 0.1% BSA in PBS) were added to each well of a 96-well plate (Greinier). Plasma at 1:40, 1:200 and 1:1,000 dilutions in PBS were added to beads and incubated at 48 °C for 18 h of shaking. Beads were washed three times with 200 µl of PBS-Tween. Individual isotype and subclass detection reagents (bulk IgM, bulk IgG, IgG1, IgG2, IgG3, IgG4, IgA1 and IgA2) conjugated to phycoerythrin (SouthernBiotech) or Fc-receptor (FcR) variants (Duke Human Vaccine Institute), biotinylated by BirA (Avidity) and conjugated to streptavidin phycoerythrin (Prozyme) following the manufacturer’s instructions, were added and incubated at room temperature for 2 h of shaking. Beads were washed three times with 200 µl PBS-Tween, resuspended in 100 µl Bio-Plex sheath fluid, and read on Bio-Plex 200. Each plasma sample was tested in duplicate (Extended data Fig. 7) across all dilutions as indicated with median fluorescence intensities (MFIs) from three dilutions used to calculate area under the curve (AUC) by GraphPad Prism v.7.0.

### Glycan arrays

The NCFG glycan v.1 glycan microarray (NCFGv1) consists of 99 different glycans that were conjugated to the bifunctional fluorescent linker AEAB prepared according to Song et al.^55^. The AEAB-labeled glycans were printed on NHS-functionalized microarray slides and assays were performed as previously described^56^. More details on the individual glycans can be found at https://ncfg.hms.harvard.edu. Briefly, plasma samples were diluted 1:50 in TSM buffer containing 0.05% Tween-20 and 1% BSA and incubated on the slides for 1 h. Slides were washed in TSM wash buffer (TSM-0.05%, Tween-20), and incubated simultaneously with secondary antibodies to IgG and IgM (Invitrogen, 5 μg ml^–1^ each) for 1 h to detect the presence of anti-glycan antibodies in the plasma. Slides were then washed, dried, imaged and analyzed. A subset of individual plasma samples at the same dilution was also run on the CFG mammalian glycan array v.5.2, which contains over 600 unique carbohydrate structures. More details on the individual glycans, methods and analysis can be found at www.functionalglycomics.org.

### Avidity ELISA

The calculated avidity of plasma anti-PPD IgG was determined as the molar concentration of urea required to reduce the initial absorbance by 50%^57^. Plasma from ‘resisters’ (*n* = 40), LTBI controls (*n*= 39) and healthy, HIV-uninfected North Americans (*n* = 10) was pooled for evaluation of IgG reactivity to PPD by ELISA in a dilution series of urea 0–7 M for 15 min before the addition of the secondary antibody. Each plasma group had evaluations performed in triplicate. The statistical significance was calculated using the Student’s *t*-test.

### IgG glycans

PPD (Statens Serum Institute) was biotinylated with Sulfo-NHS-LC-LC Biotin (Thermo Scientific) and excess biotin was removed with a 3-kDa molecular mass cutoff column (Amicon/EMD, UFC500396) following manufacturers’ instructions. Biotinylated PPD (20 μg per individual sample) was coupled to streptavidin magnetic beads (New England BioLabs, S1420S) (50 μl per individual sample) rotating for 1 h at room temperature following the manufacturer’s instructions. PPD-coupled streptavidin magnetic beads were washed with 0.5 M NaCl, 20 mM Tris-HCl (pH 7.5) and 1 mM ethylenediaminetetraacetic acid five times. Plasma (200 μl) from individuals was blocked with non-coated streptavidin magnetic beads (50 μl per individual sample) for 2 h at room temperature. PPD-adsorbed streptavidin magnetic beads and blocked plasma were incubated subsequently for 2 h at room temperature while rotating. Antibodies bound to PPD-coupled streptavidin beads were pelleted with a magnet and any supernatant removed. Non-antigen-specific bulk IgG was purified from 5 μl of plasma per individual with protein G beads (Millipore PureProteome, LSKMAGG10) following the manufacturer’s instructions and washed in PBS-Tween three times. Fc from antigen-specific antibody bound to PPD-coupled streptavidin beads or bulk IgG bound to protein G beads was cleaved via IdeZ (New England Biolabs, P0770S) following the manufacturer’s instructions. The subsequent supernatant containing antibody Fc was removed and glycans isolated and labeled with Glycan Assure APTS kit (Life Technologies, A28676) following the manufacturer’s instructions. PPD-specific IgG glycans were run with a LIZ 600 DNA ladder in Hi-Di formamide (ThermoFisher) on an ABI 3130XLl DNA sequencer. Data were analyzed using GeneMapper v.5.0. Bulk IgG glycans were run on Applied Biosystems 3500/3500xL Genetic Analyzer and analyzed with GlycanAssure Data Acquisition Software v.1.1.

### Intracellular cytokine staining

ICS staining was performed on 25 ‘resisters’ and 25 LTBI controls, and samples were processed in two batches in which the number of ‘resisters’ and controls was matched. PBMCs were thawed and washed in warm, sterile-filtered RPMI 1640 (Gibco) supplemented with 10% fetal bovine serum (FBS) (HyClone) and 2 μl ml^–1^ Benzonase (Millipore) and enumerated using the Guava easyCyte (Millipore) with guavaSoft v.2.6 software. PBMCs were then resuspended in a 50 ml conical flask at a density of 2 × 10^6^ cells ml^–1^ in RPMI/10% FBS and allowed to rest overnight at 37 °C in humidified incubators supplemented with 5% CO_2_. The following day, the PBMCs were enumerated using the Guava easyCyte and resuspended at a density of 5 × 10^6^ cells ml^–1^. To observe ICS after antigen stimulation, 1 × 10^6^ cells per well were plated into a 96-well U-bottomed plate and stimulated in the presence of peptide pools, 100 μg ml^–1^ *Mtb* Whole Cell Lysate, H37Rv (BEI Resources), 0.25 μg ml^–1^ SEB (List Biological Laboratories, Inc.) or 0.5% DMSO (Sigma). Peptide Pool 1 consisted of ESAT6 and CFP10 (BEI Resources), and Peptide Pool 2 consisted of Ag85A, Ag85B and TB10.4 (BEI Resources), with final concentrations of each peptide at 1 μg ml^–1^. In addition to antigen, each stimulation cocktail consisted of 1 μg ml^–1^ anti-CD28/49d (BD Biosciences), 10 μg ml^–1^ Brefeldin A (Sigma), GolgiStop (BD Biosciences), prepared according to manufacturer’s instructions, and anti-CD107a phycoerythrin Cy7 (BD Biosciences). Stimulation and all remaining steps were performed in the dark. The cell mixture was allowed to incubate for 6 h at 37 °C/5% CO_2_ after which ethylenediaminetetraacetic acid (ThermoFisher Scientific), at a final concentration of 2 mM, was added to disaggregate cells. Samples were stored overnight at 4 °C and then stained and acquired by flow cytometry the following day. We used a previously published optimized and validated 12-color panel^26,27^ to examine cells (see Supplementary Table 4). Briefly, cells were first washed twice in PBS (Gibco) and then stained for 20 min at room temperature with AViD Live/Dead viability dye (Life Technologies), prepared according to the manufacturer’s instructions. After washing twice with PBS, the cells were incubated with 1× FACS Lyse (BD Biosciences) at room temperature for 10 min, washed once with FACS buffer (1× PBS supplemented with 0.2% BSA (Sigma)), and then incubated again at room temperature for 10 min with 1× FACS Perm II (BD Biosciences). The cells were washed twice with FACS buffer and then stained for the remaining markers: CD3 ECD, CD4 APC Ax750 (Beckman Coulter), CD8 PerCP Cy5.5, IFN-γ V450, TNF FITC, IL-2 phycoerythrin, IL-4 APC, CD40L phycoerythrin Cy5 (BD Biosciences) and IL-17a Ax700 (BioLegend). The choice of T cell and functional markers was determined by those included in a formally validated endpoint assay for vaccine studies^26,27^. After two final washes in FACS buffer, the cells were fixed in 1% paraformaldehyde (Electron Microscopy Sciences) in PBS and acquired on a BD LSRFortessa (BD Biosciences), equipped with a high-throughput sampler and configured with blue (488 nm), green (532 nm), red (628 nm), violet (405 nm) and ultraviolet (355 nm) lasers using standardized good clinical laboratory practice procedures to minimize the variability of data generated.

### In vitro macrophage *Mtb* survival

CD14-positive cells were isolated from whole blood from HIV-seronegative donors using the EasySep CD14 Selection Kit II following the manufacturer’s instructions (Stem Cell Technologies) and matured for 7 days in RPMI (Invitrogen) and 10% FBS (Life Technologies) in low-adherent flasks (Corning). Monocyte-derived macrophages (5 × 10^4^ per well) were plated in glass-bottomed, 96-well plates (Greiner) 24 h before *Mtb* infection. Live dead reporter bacteria constitutively expressing mCherry and a tetracycline-inducible green fluorescent protein (GFP)^58^ were cultured in 0.5 mg ml^–1^ Hygromycin 7H9 media (BD Biosciences) at 37 °C in log phase, washed, sonicated and passed through a 5-mm filter (Milliplex) to obtain a single cell suspension before infection at multiplicity of infection 1 for 14 h at 37 °C. Extracellular bacteria were washed off and purified IgG at 0.1 mg ml^–1^, 0.01 mg ml^–1^ and 0.001 mg ml^–1^ was added. After 3 days of treatment, anhydrotetracycline (Sigma) (200 ng ml^–1^) was added for 16 h to induce GFP expression in live but not dead bacteria. The cells were washed, fixed and stained with DAPI. Images were obtained via an Operetta High-Content Imaging Fluorescence Microscope (Perkin-Elmer) outfitted with a 20× NA objective. The total *Mtb* bacterial burden was determined based on mCherry^+^ pixels. Transcriptionally active *Mtb* bacterial burden was determined based on GFP^+^ pixels. Data from technical triplicates per donor were analyzed using CellProfiler v.3.1.8 (ref. ^17,59^). Bacterial survival was calculated as a ratio of live to total bacteria (the number of GFP^+^ pixels (live) divided by the number of mCherry^+^ pixels (total burden)).

### IL-1β ELISA

Human IL-1β levels in the culture supernatants from the in vitro macrophage assays were determined using a Human IL-1β High sensitivity ELISA (eBioscience). The ratio of the level of IL-1β in the presence of ‘resister’ or LTBI IgG to the absence of antibodies was used to calculate the relative IL-1β level (‘resister’ or LTBI/no IgG).

### Fc functional assays

#### Antibody-dependent cellular phagocytosis

**Extended Data Fig. 8:**
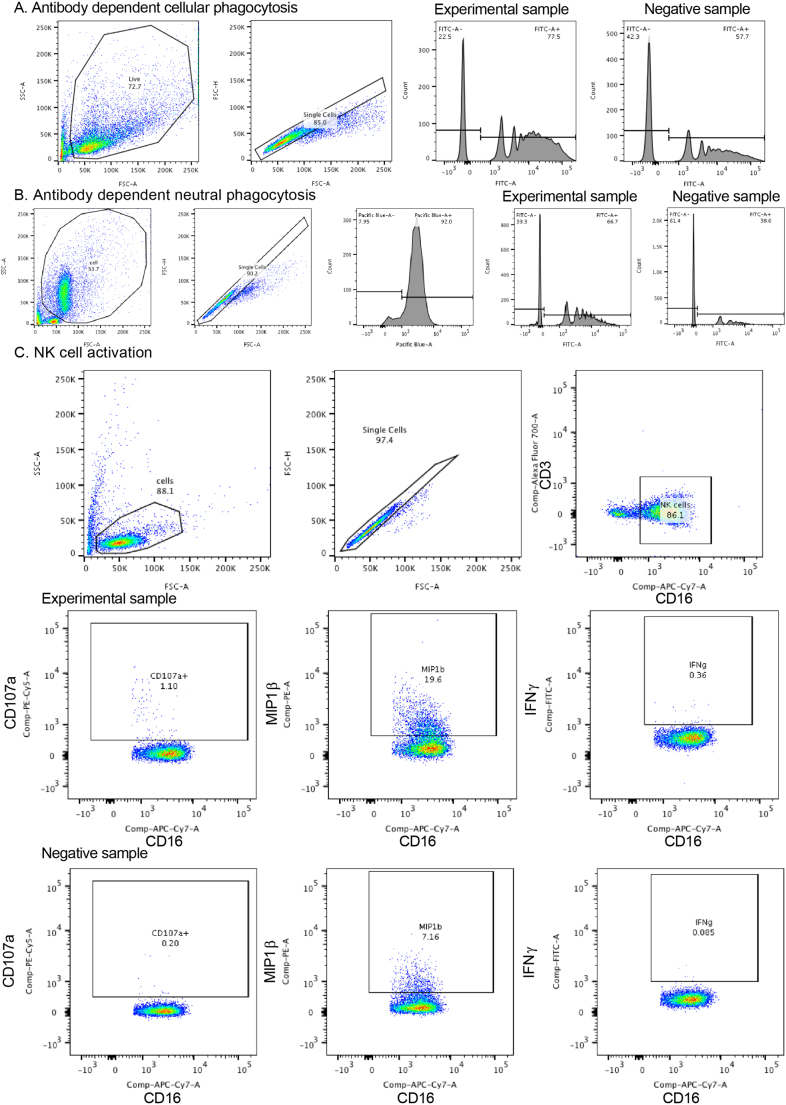
Gating strategies for antibody-dependent phagocytosis, antibody-dependent neutrophil phagocytosis and NK cell activation. **a**, Antibody-dependent cellular phagocytosis of PPD and or ESAT6/CFP10 adsorbed fluorescent FITC beads was measured in THP-1 monocytes. After gating on size, granularity and singlets, the frequency and mean fluorescence intensity of FITC beads was measured. The phagocytic score was calculated as the integrated MFI (percentage frequency × MFI/10,000)^50^. **b**, Antibody-dependent neutrophil phagocytosis of PPD adsorbed fluorescent FITC beads was measured. Neutrophils were identified by CD66b staining after gating on size, granularity and singlets. The frequency and mean fluorescence intensity of FITC beads was measured, and phagocytic score was calculated as the integrated MFI (percentage frequency × MFI/10,000). **c**, For antibody-dependent NK-cell activation, cells were first captured after gating on size, granularity and singlets. CD3+ T lymphocytes were gated out and CD16 was used to identify NK cells for which MIP-1β expression, and CD107a and IFN-γ production were measured.

THP-1 cell phagocytosis of antigen-coated beads was conducted as previously described^17,60^. *Mtb* antigens were biotinylated with Sulfo-NHS-LC Biotin (ThermoFisher) following the manufacturer’s instructions and incubated with 1-μm fluorescent neutravidin beads (Invitrogen) at 4 °C for 16 h. Excess antigen was washed away. Antigen-coated beads were incubated with plasma (at 1:100, 1:1,000, 1:10,000 dilutions in PBS) for 2 h at 37 °C. THP-1 cells (1 × 10^5^ per well) were added and incubated at 37 °C for 16 h. Bead uptake was measured in fixed cells using flow cytometry on a BD LSRII (BD Biosciences) equipped with a high-throughput sampler. Phagocytic scores are presented as the integrated MFI (percentage bead-positive frequency × MFI per 10,000) (Extended data Fig. 8a)^50^. Antibody-dependent cellular phagocytosis experiments for individual plasma samples were performed in duplicate in two independent experiments.

#### Antibody-dependent neutrophil phagocytosis

Whole healthy donor blood was mixed with an equal volume of 3% Dextran-500 (ThermoFisher) and incubated for 25 min at room temperature to lyse and pellet the red blood cells. Leukocytes were removed and washed in Hanks’ balanced salt solution without calcium and magnesium (ThermoFisher), separated using Ficoll-Histopaque (Sigma-Aldrich) centrifugation and washed with PBS. PPD-conjugated beads, as described above, were incubated with plasma (at 1:30, 1:100, 1:1,000, 1:10,000 dilutions in PBS) for 2 h at 37 °C. Isolated neutrophils (1 × 10^5^ per well) were added and incubated for 16 h at 37 °C. Bead uptake was measured as described above. The purity of the neutrophils was confirmed by staining with CD66b (BioLegend). Phagocytic scores are presented as the integrated MFI (percentage bead-positive frequency × MFI per 10,000) (Extended data Fig. 8b). Antibody-dependent neutrophil phagocytosis experiments for individual plasma samples were performed in duplicate across dilutions using cells from five healthy HIV-negative donors.

#### NK cell activation

ELISA-based, antibody-dependent, NK-cell activation assays were performed^17,61^. ELISA plates (ThermoFisher NUNC MaxiSorp flat bottom) were coated with PPD (300 ng per well) or BSA as a negative control at 4 °C for 16 h. Plasma (at 1:100, 1:1,000, 1:10,000 dilutions in PBS) was added to each well. NK cells were isolated from whole blood from healthy HIV-negative donors with RosetteSep (Stem Cell Technologies). NK cells (5 × 10^4^ per well), anti-CD107a-phycoerythrin-Cy5 (BD Biosciences), Brefeldin A (10 mg ml^–1^) (Sigma) and GolgiStop (BD Biosciences) were added and incubated for 5 h at 37 °C. Cells were stained for surface markers using anti-CD16–allophycocyanin-Cy7 (BD), anti-CD56-phycoerythrin-Cy7 (BD) and anti-CD3-AlexaFluor 700 (BD Biosciences), and intracellularly with anti-IFN-γ-APC (BD Biosciences) and anti-macrophage inflammatory protein-1β-phycoerythrin (BD Biosciences) using Fix and Perm A and B solutions (ThermoFisher). NK cells were defined as CD3^–^ and CD16/56^+^ (Extended data Fig. 8c). NK-cell activation assays were performed across the dilutions stated above using cells from four healthy HIV-negative donors.

### Computational/statistics

To estimate the incidence rate of TB, we calculated the person-years of follow-up using the last visit date during the phase 1 study^9^, and the date when we re-traced them during the re-tracing study. Among the 144 PTST– and 303 TST-positive individuals, there were 6 who developed TB from the end of the phase 1 study; these events and their person-years were used to calculate incidence.

LASSO was used initially to reduce highly correlated features, with the goal of selecting the minimal number of individual antibody features that captured the overall variation among the ‘resisters’ and control subjects. PLSDA was then used to visualize antibody profiles, using these minimal LASSO-selected features, in multivariate space. To estimate the minimal correlates that best explain group differences without overfitting, 5,000 repeated, 10-fold nested, cross-validation was designed. In each repetition, the dataset was randomly divided into groups of 10 arbitrarily assorted individuals, where 90% of the dataset was used to build the model and the remaining holdout set was used to test the model prediction, and the goodness of fit of the model was measured by classification accuracy between ‘resisters’ and LTBI controls. Ultimately, this approach resulted in the generation of a model with the minimal set of features that generates the best classification prediction in a cross-validation test. In addition, variable importance in projection, using a weighted sum of squares of the PLSDA weights to summarize the importance of individual selected features from the PLSDA model, was also computed. To estimate the statistical significance of the optimized model with the defined correlates, we employed two types of permutation tests—(1) shuffling the outcome label and (2) selecting the randomized correlates—to test the likelihood of obtaining a model prediction accuracy (displaying in a receiver operating characteristic curve) by chance. Each permutation test was performed 1,000 times to generate an empirical null distribution and an exact *P* value of the correct model was computed. All data used in this analysis are available in the accompanying dataset.

**Extended Data Fig. 9:**
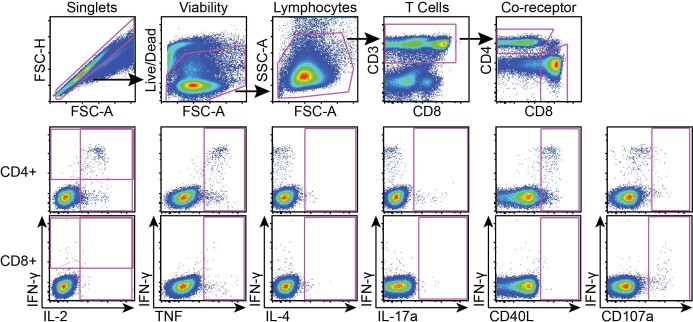
Gating strategy for ICS. A representative gating tree for flow analysis of cells following stimulation with antigen. Gating (top row) began with singlets, followed by viable cells. Lymphocytes were then identified by size, and CD3 expression was used to focus on T cells. The T cells were then separated into CD4 versus CD8 co-receptor subsets. For each subset (CD4 middle row, CD8 bottom row), cytokines were visualized by gating against IFN-γ. Pink boxes demonstrated positive staining. Positivity of cytokines was determined by DMSO negative and SEB positive controls as well as by gating on CD3 negative populations (not shown). Gating for CD4 and CD8 cytokines was determined separately.

The raw ICS data were compensated for and manually gated using FlowJo (TreeStar Inc.). A representative gating tree is shown in Extended data Fig. 9. The data were then processed using the OpenCyto framework in the R programming environment^62^. Although we began with 25 subjects for each group, samples with poor viability defined on the basis of low CD3 counts (<10,000 cells) or low CD4 counts (<3,000 cells) were excluded from further analysis. The final data analysis included the following: Peptide Pools 1 and 2 consisted of 22 ‘resisters’ and 19 LTBI controls, *Mtb* lysate consisted of 21 ‘resisters’ and 20 LTBI controls, and SEB consisted of 22 ‘resisters’ and 20 LTBI controls. Total event counts ranged from 49,252 events to 584,133 events per sample, and from 15,183 events to 143,933 events per sample for CD3. To analyze which T cell subsets were being activated by the various stimulations, we used COMPASS^28^. COMPASS uses a Bayesian computational framework to identify T cell subsets for which there is a high probability of an antigen-specific response. For each combination of subset and patient, COMPASS compared the proportion of gated events in the antigen-stimulated sample with the proportion of gated events in the control sample. Notably, COMPASS reported only the probability of detecting T cell responses with a particular functional profile, rather than the frequency, which was calculated separately. For a given subject, COMPASS was also used to compute a functionality score that summarized the entire functionality profile into a single number. For the data presented here, COMPASS was applied to each of the five stimulations of CD4^+^ T cells. Each one of the analyses was unbiased and considered all of the 128 possible cytokine functions (defined as a Boolean combination). Individuals with a high probability of response across many subsets were accordingly assigned a high functionality score. Magnitudes of T cell responses were calculated independent of COMPASS as the maximum of zero, or the proportion of gated events in the stimulated condition minus the proportion of gated events in the unstimulated condition. Note that two subjects can have equally high probabilities of response for a given subset, even if one patient’s background-corrected proportion of gated events is higher than the other’s. All the flow cytometry data are available for download from ImmPort (www.immport.org) under study accessoin SDY1385, ‘Flow cytometry of T cells for TB Resistance Study’. The code to complete COMPASS analyses can be found at https://github.com/seshadrilab/ResisterCOMPASSAnalysis.

### Reporting Summary

Further information on research design is available in the Nature Research Reporting Summary linked to this article.

## Online content

Any methods, additional references, Nature Research reporting summaries, source data, statements of code and data availability and associated accession codes are available at 10.1038/s41591-019-0441-3.

## Supplementary Information

Supplementary Information Supplementary Tables 1–4

Reporting Summary

Supplementary Data Supplementary data including demographics, clinical characteristics, antibody, and T cell data.

## Data Availability

The data supporting the findings of this study are available in the accompanying Supplmentary Information, from ImmPort (www.immport.org) under study accessoin SDY1385 ‘Flow cytometry of T cells for TB Resistance Study’, and from the corresponding author upon reasonable request.
