# Publisher Correction: IFN-γ-independent immune markers of *Mycobacterium tuberculosis* exposure

**DOI:** 10.1038/s41591-019-0519-y

**Published:** 2019-06-20

**Authors:** Lenette L. Lu, Malisa T. Smith, Krystle K. Q. Yu, Corinne Luedemann, Todd J. Suscovich, Patricia S. Grace, Adam Cain, Wen Han Yu, Tanya R. McKitrick, Douglas Lauffenburger, Richard D. Cummings, Harriet Mayanja-Kizza, Thomas R. Hawn, W. Henry Boom, Catherine M. Stein, Sarah M. Fortune, Chetan Seshadri, Galit Alter

**Affiliations:** 1 Department of Immunology and Infectious Diseases, Harvard TH Chan School of Public Health, Boston, MA USA; 2 Ragon Institute of MGH, MIT and Harvard, Cambridge, MA USA; 3 Department of Medicine, University of Washington, Seattle, WA USA; 4 Department of Biological Engineering, MIT, Cambridge, MA USA; 5 Department of Surgery, Beth Israel Deaconess Medical Center, Harvard Medical School, Boston, MA USA; 6 Department of Medicine, Makerere University, Kampala, Uganda; 7 Department of Medicine, Case Western Reserve University and Univ. Hospitals Cleveland Medical Center, Cleveland, OH USA; 8 Department of Population & Quantitative Health Sciences, Case Western Reserve University, Cleveland, OH USA

**Keywords:** Cellular immunity, Biomarkers, Tuberculosis, Antibodies

Correction to: *Nature Medicine* 10.1038/s41591-019-0441-3, published online 20 May 2019.

In the version of this article originally published, there was an error in the abstract. The word disease should not have been included in the sentence “These individuals were highly exposed to Mtb but tested negative disease by IFN-γ release assay and tuberculin skin test, ‘resisting’ development of classic LTBI”. The sentence should have been “These individuals were highly exposed to Mtb but tested negative by IFN-γ release assay and tuberculin skin test, ‘resisting’ development of classic LTBI.” The error has been corrected in the HTML and PDF versions of this article.

