# Supplementary material for: IFN-γ-independent immune markers of Mycobacterium tuberculosis exposure
Source: Nat Med. 2019 May 20;25(6):977–87. doi: 10.1038/s41591-019-0441-3 (PMC6559862; doi:10.1038/s41591-019-0441-3)
Supplement: Supplementary file 1 — Supplementary Tables 1–4 [file 41591_2019_441_MOESM1_ESM.pdf]

nature medicine

SUPPLEMENTARY INFORMATION

https://doi.org/10.1038/s41591-019-0441-3

In the format provided by the authors and unedited.

IFN-γ-independent immune markers of *Mycobacterium tuberculosis* exposure

Lenette L. Lu[1,2], Malisa T. Smith[3], Krystle K. Q. Yu[3], Corinne Luedemann[2], Todd J. Suscovich[2], Patricia S. Grace[2], Adam Cain[2], Wen Han Yu[2,4], Tanya R. McKitrick[5], Douglas Lauffenburger[4], Richard D. Cummings[5], Harriet Mayanja-Kizza[6], Thomas R. Hawn[3], W. Henry Boom[7], Catherine M. Stein[7,8], Sarah M. Fortune[1,2], Chetan Seshadri[3,9]* and Galit Alter[2,9]*

[1]Department of Immunology and Infectious Diseases, Harvard TH Chan School of Public Health, Boston, MA, USA. [2]Ragon Institute of MGH, MIT and Harvard, Cambridge, MA, USA. [3]Department of Medicine, University of Washington, Seattle, WA, USA. [4]Department of Biological Engineering, MIT, Cambridge, MA, USA. [5]Department of Surgery, Beth Israel Deaconess Medical Center, Harvard Medical School, Boston, MA, USA. [6]Department of Medicine, Makerere University, Kampala, Uganda. [7]Department of Medicine, Case Western Reserve University and Univ. Hospitals Cleveland Medical Center, Cleveland, OH, USA. [8]Department of Population & Quantitative Health Sciences, Case Western Reserve University, Cleveland, OH, USA. [9]These authors contributed equally: Chetan Seshadri, Galit Alter. *e-mail: seshadri@u.washington.edu; galter@mgh.harvard.edu

Supplementary Table 1: Demographic characteristics of study group.

Risk score is determined as previously described[16]. Means and medians are presented as indicated. Categorical variables were compared using a chi-square test, and continuous variables were compared using a Mann-Whitney test; p-values indicate significance according to these statistics. BCG scar indicates prior vaccination with Bacillus Calmette–Guérin (BCG).

	"resister" TST-/IGRA-	LTBI TST+/IGRA+	p-value
**Current age (median)**	20.00	22.50	0.371
**Age at recruitment (median)**	13	13.5	0.502
**Sex (% Female)**	42.9%	43.5%	0.951
**% with BCG scar**	61.2%	65.2%	0.830
**Pediatric risk score (mean)**	6.07	6.35	0.113
**Adult risk score (mean)**	6.26	6.70	0.296
**Duration of follow-up in years (median)**	9.31	8.47	0.780
**BMI (median)**	20.92	21.83	0.388

Supplementary Table 2: Demographics of subjects included in this study compared to those from the larger "resister" cohort.

Risk score is determined as previously described[16]. Means and medians are presented as indicated. Categorical variables were compared using a chi-square test, and continuous variables were compared using a Mann-Whitney test; p-values indicate significance according to these statistics. BCG scar indicates Bacillus Calmette–Guérin prior vaccination.

	Subjects in this study	Subjects not included in this study	p-value
**Current age (median)**	21.00	22.00	0.762
**Sex (% Female)**	43.2%	50.8%	0.226
**% with BCG scar**	63.2%	69.1%	0.972
**Pediatric risk score (mean)**	6.20	6.15	0.998
**Adult risk score (mean)**	6.49	6.45	0.718
**Duration of follow-up in years (median)**	9.16	9.99	0.024

Supplementary Table 3: Incidence of new TB cases
Person-years of follow-up were calculated based on last visit and subsequent follow-up dates. Number of new TB cases are shown along with the calculated incidence rate and 95% confidence intervals (CI).

	PTST-<br>TST-/IGRA-	LTBI<br>TST+/IGRA+
**Total cases**	144	303
**Person-years of follow-up**	1020	2212
**Cases of active TB**	2	4
**Incidence rate [95% CI]**	0.00196 [0-0.0047]	0.00180 [0.000036-0.00357]

Supplementary Table 4: Multi-parameter flow cytometry panel. The results reported in Figures 3 and 4 of this manuscript utilized an 11-color flow cytometry panel to analyze T cells following in vitro stimulation. Details regarding the antibodies used for these experiments are provided here.

Specificity	Fluorophore	Clone	Catalog Number	Lot Number	Supplier
**TNF**	FITC	MAb11	554512	6245784	BD Biosciences
**CD8a**	PerCP Cy5.5	SK1	341051	6012504	BD Biosciences
**IL-2**	PE	MQ1-17H12	559334	3263570	BD Biosciences
**CD3**	ECD	UCHT1	IM2705U	100	Beckman Coulter
**CD40L**	PE Cy5	TRAP1	555701	6119902	BD Biosciences
**CD107a**	PE Cy7	H4A3	561348	6084604	BD Biosciences
**IL-4**	APC	MP4-25D2	554486	5234935	BD Biosciences
**IL-17a**	Ax700	BL168	512318	B218394	BioLegend
**CD4**	APC Ax750	13B8.2	A94685	36	Beckman Coulter
**IFN-γ**	V450	B27	560371	6105971	BD Biosciences
**Dead Cell Stain**	AmCyan		L34957	1878891	Life Technologies
