# Supplementary material for: IFN-γ-independent immune markers of Mycobacterium tuberculosis exposure
Source: Nat Med. 2019 May 20;25(6):977–87. doi: 10.1038/s41591-019-0441-3 (PMC6559862; doi:10.1038/s41591-019-0441-3)
Supplement: Supplementary file 2 — Reporting Summary [file 41591_2019_441_MOESM2_ESM.pdf]

nature research

Corresponding author(s): Galit Alter

Reporting Summary

Nature Research wishes to improve the reproducibility of the work that we publish. This form provides structure for consistency and transparency in reporting. For further information on Nature Research policies, see Authors & Referees and the Editorial Policy Checklist.

Statistical parameters

When statistical analyses are reported, confirm that the following items are present in the relevant location (e.g. figure legend, table legend, main text, or Methods section).

n/a	Confirmed	
☐	☒	The exact sample size (n) for each experimental group/condition, given as a discrete number and unit of measurement
☐	☒	An indication of whether measurements were taken from distinct samples or whether the same sample was measured repeatedly
☐	☒	The statistical test(s) used AND whether they are one- or two-sided<br>*Only common tests should be described solely by name; describe more complex techniques in the Methods section.*
☐	☒	A description of all covariates tested
☐	☒	A description of any assumptions or corrections, such as tests of normality and adjustment for multiple comparisons
☐	☒	A full description of the statistics including central tendency (e.g. means) or other basic estimates (e.g. regression coefficient) AND variation (e.g. standard deviation) or associated estimates of uncertainty (e.g. confidence intervals)
☐	☒	For null hypothesis testing, the test statistic (e.g. F, t, r) with confidence intervals, effect sizes, degrees of freedom and P value noted<br>*Give P values as exact values whenever suitable.*
☐	☒	For Bayesian analysis, information on the choice of priors and Markov chain Monte Carlo settings
☐	☒	For hierarchical and complex designs, identification of the appropriate level for tests and full reporting of outcomes
☒	☐	Estimates of effect sizes (e.g. Cohen's d, Pearson's r), indicating how they were calculated
☐	☒	Clearly defined error bars<br>*State explicitly what error bars represent (e.g. SD, SE, CI)*

Our web collection on statistics for biologists may be useful.

Software and code

Policy information about availability of computer code

Data collection	Flow cytometry data was analyzed using FlowJo 10.
Data analysis	Prism 8.0.2 and Matlab R2018b with customized code were used for antibody data analysis. CellProfiler 3.1.8 was used for image analysis. Glycan data was analyzed using GeneMapper 5.0 or GlycanAssure™ Data Acquisition Software 1.0 as noted. From PBMC, cells were enumerated using the Guava easyCyte with guavaSoft 2.6 software. T cell data was processed using using the OpenCyto framework in the R programming environment. To analyze which T-cell subsets were being activated by the various stimulations, COMbinatorial Polyfunctionality Analysis of Antigen-Specific T cell Subsets (COMPASS) was used as as detailed in the manuscript and also previously described (Lin et al 2015). Code to complete COMPASS analyses can be found at https://github.com/seshadrilab/ResisterCOMPASSAnalysis. Details regarding data analysis for glycan arrays are found at https://www.functionalglycomics.org.

For manuscripts utilizing custom algorithms or software that are central to the research but not yet described in published literature, software must be made available to editors/reviewers upon request. We strongly encourage code deposition in a community repository (e.g. GitHub). See the Nature Research guidelines for submitting code & software for further information.

Data

Policy information about availability of data

All manuscripts must include a data availability statement. This statement should provide the following information, where applicable:

- Accession codes, unique identifiers, or web links for publicly available datasets
- A list of figures that have associated raw data
- A description of any restrictions on data availability

The data that support the findings of this study are available from the corresponding author upon reasonable request. All antibody data used is available in the accompanying Supplemental Table. All T cell the flow cytometry data are available for download from ImmPort (www.immport.org/TBD). Code to complete COMPASS analyses can be found at https://github.com/seshadrilab/ResisterCOMPASSAnalysis."

Field-specific reporting

Please select the best fit for your research. If you are not sure, read the appropriate sections before making your selection.

☒ Life sciences ☐ Behavioural & social sciences ☐ Ecological, evolutionary & environmental sciences

For a reference copy of the document with all sections, see nature.com/authors/policies/ReportingSummary-flat.pdf

Life sciences study design

All studies must disclose on these points even when the disclosure is negative.

Sample size	Sample size for antibody studies were based on prior published studies of individuals with latent and active TB (Lu et al 2016). Sample size for T cell studies were motivated by balancing for confounders as well as recently published study of South African adolescents (Seshadri et al 2015).
Data exclusions	Pre established criteria led to the exclusion of one individual from the LTBI group due to clinical classification. For T cell studies samples with poor viability defined on pre established criteria of low CD3 counts (<10,000 cells) or low CD4 counts (<3,000 cells) were excluded from further analysis.
Replication	Luminex data was gathered twice in technical duplicate across three different plasma dilutions. Fc effector functional assays were performed in technical duplicate across three different plasma dilutions. For antibody Fc functional assays involving primary immune cells, four different donors were used. For primary macrophage assays involving Mycobacterium tuberculosis, three different donors were used and each condition was performed in triplicate over three dilutions. Five additional macrophage donors were used at one dilution given limitations of sample availability. Replicates in all assays were confirmatory and the extent described within the text and shown in the main figures,.
Randomization	Individuals were allocated into experimental groups by clinical criteria as described in the manuscript with matching based on age, gender, and epidemiological risk factors.
Blinding	Luminex, Fc effector functions, IgG glycosylation data was gathered blinded. Analysis was performed after collection of aforementioned data and unblinding.

Reporting for specific materials, systems and methods

Materials & experimental systems

n/a	Involved in the study	
☐	☒	Unique biological materials
☐	☒	Antibodies
☐	☒	Eukaryotic cell lines
☒	☐	Palaeontology
☒	☐	Animals and other organisms
☐	☒	Human research participants

Methods

n/a	Involved in the study	
☒	☐	ChIP-seq
☐	☒	Flow cytometry
☒	☐	MRI-based neuroimaging

Unique biological materials

Policy information about availability of materials

Obtaining unique materials	All reagents are commercially available other than the patient plasma and cell samples.

Antibodies

Antibodies used

Commercial antibodies used in this study are described in materials and methods with supplier name and catalog number and are listed below. The generation of non commercial antibody samples is described in the materials and methods.

Specificity	Fluorophore	Clone	Catalog #	Lot #	Supplier
TNF	FITC	MAb11	554512	6245784	BD Biosciences
CD8a	PerCP Cy5.5	SK1	341051	6012504	BD Biosciences
IL-2	PE	MQ1-17H12	559334	3263570	BD Biosciences
CD3	ECD	UCHT1	IM2705U	100	Beckman Coulter
CD40L	PE Cy5	TRAP1	555701	6119902	BD Biosciences
CD107a	PE Cy7	H4A3	561348	6084604	BD Biosciences
IL-4	APC	MP4-25D2	554486	5234935	BD Biosciences
IL-17a	Ax700	BL168	512318	B218394	BioLegend
CD4	APC Ax750	13B8.2	A94685	36	Beckman Coulter
IFN-γ	V450	B27	560371	6105971	BD Biosciences
Dead Cell Stain	AmCyan		L34957	1878891	Life Technologies
CD107a	PE-Cy5	H4A3	555802	396136	BD Biosciences
CD16	APC-Cy7	3G8	557758	396864	BD Biosciences
CD56	PE-Cy7	B159	557747	396853	BD Biosciences
CD3	Alexa Fluor 700	UCHT1	557943	396952	BD Biosciences
IFN-γ	APC	B27	554702	398580	BD Biosciences
MIP-1b	PE	D21-1351	550078	393549	BD Biosciences
CD66b	Pacific Blue	G10F5	305112	2563294	Biolegend
IgG4 Fc	PE	HP6025	9200-09	B5313-SP55B	SouthernBiotech
IgG3 Hinge	PE	HP6050	9210-09	F1818-Y198	SouthernBiotech
IgG2 Fc	PE	HP6002	9070-09	B3016-T066Y	SouthernBiotech
IgG1 Hinge	PE	4E3	9052-09	G4015-Z678	SouthernBiotech
IgG Fc	PE	JDC-10	9040-09	A1918-Z368	SouthernBiotech
IgM	PE	SA-DA4	9020-09	C2718-T798B	SouthernBiotech
IgA1	PE	B3506B4	9130-09	C3818-S378C	SouthernBiotech
IgA2	PE	A9604D2	9140-09	J2113-S498B	SouthernBiotech
IgG Fc	Alexa Fluor 488	polyclonal	109-545-008	126214	Jackson Immunoresearch
IgM Fc5μ	Alexa Fluor 647	polyclonal	109-605-043	128711	Jackson Immunoresearch

Validation

References are included for manuscripts by the authors that have validated all reagents used for the analysis described in this manuscript. More specifically, for antibody dependent neutrophil phagocytosis, validation of CD66b antibody is described in 1) Lu et al 2016 Cell PMID 27667685, 2) Worley et al 2018 Journal of Immunological Methods PMID 29605231, 3) Sapphire et al 2018, Cell PMID 30096313, 4) Gunn et al 2018 Cell Host and Microbe PMID 30092199 5) Gilchuk et al 2018 Immunity PMID 30029854, 6) Bornholdt et al 2019 Cell Host and Microbe PMID 30629918. Validation of antibodies for antibody dependent NK cell activation have been described in 1) Jegaskanda et al 2013 J Virology PMID 23468501, 2) Chung et al 2014 Sci Transl Med 24648341, 3) Ackerman et al 2016 PMID 26745376 , 4) Lu et al 2016 Cell PMID 27667685, 5) Sapphire et al 2018, Cell PMID 30096313, 6) Gunn et al 2018 Cell Host and Microbe PMID 30092199 7) Gilchuk et al 2018 Immunity PMID 30029854. The choice of T cell and functional markers was determined by those included in a formally validated endpoint assay for vaccine studies: Horton et al 2007 J Immunol Methods PMID 17451739 and De Rosa et al 2012 Cytometry A PMID 23081852.

Eukaryotic cell lines

Policy information about cell lines

Cell line source(s)	THP1 from ATCC Cat#: TIB202
Authentication	Authentication was performed via STR Profiling service ATCC 135-XV
Mycoplasma contamination	THP1 cell line was tested and negative for mycoplasma via ATCC Univ Mycoplasma kit 30-1012K
Commonly misidentified lines (See ICLAC register)	No commonly misidentified cell lines were used.

Human research participants

Policy information about studies involving human research participants

Population characteristics

Relevant covariate population characteristics of age, gender, TB diagnosis, BCG vaccination, body mass index, and epidemiological risk score are reported in Extended data Tables 1 and 3 and in brief summarized below:

	“resister” (TST-/IGRA- LTBI)	vs TST+/IGRA+	p-value
Current age (median)	20.00	22.50	0.371
Age at recruitment (median)	13	13.5	0.502
Sex (% Female)	42.9%	43.5%	0.951
% with BCG scar	61.2%	65.2%	0.830
Pediatric risk score (mean)	6.07	6.35	0.113
Adult risk score (mean)	6.26	6.70	0.296
BMI (median)	20.92	21.83	0.388

In addition, these characteristics are included in an Excel sheet provided as supplementary information. For control healthy HIV negative individuals from North America, no demographic characteristics were used for selection. Rather, selection was based on no clinical signs of illness and diagnostic testing negative for active HIV, HCV, and HBV infections.

Recruitment

Participants were recruited from the Kawempe Community Health study described in detail in Stein et al 2018 and Chheng et al 2015. In brief, adults with pulmonary TB were recruited from clinics at the Uganda National TB and Leprosy Program treatment center at Mulago Hospital, referred to the TB research clinic at Mulago Hospital, or recruited through community sensitization efforts in the Kawempe division of Kampala. As such, selection bias is primarily driven by referral to clinical care for which an effort was made to recruit through community sensitization. Houshold contacts were recruited and evaluated for TB by clinical signs and symptoms, radiology, microbiology by trained clinicians. Latent TB and "resister" phenotypes were identified by repeated testing of TST over two years of followup. To specifically determine long term outcomes of these individuals, contacts who remained persistently TST negative and contacts with traditional LTBI with equivalent baseline clinical and epidemiological risk scores were re-traced in 2014-2017 at an average of 9.5 years after initial Mtb exposure. Three sequential IGRAs and one additional TST was performed at the end of the retracing study to robustly classify LTBI and "resister" phenotypes.

Flow Cytometry

Plots

Confirm that:

☒ The axis labels state the marker and fluorochrome used (e.g. CD4-FITC).

☒ The axis scales are clearly visible. Include numbers along axes only for bottom left plot of group (a 'group' is an analysis of identical markers).

☒ All plots are contour plots with outliers or pseudocolor plots.

☒ A numerical value for number of cells or percentage (with statistics) is provided.

Methodology

Sample preparation

Peripheral blood was obtained in sodium heparin Vacutainer tubes (BD Biosciences). Plasma and PBMC were isolated by Ficoll-Histopaque (Sigma-Aldrich) density centrifugation and stored via liquid nitrogen until further use.

Instrument

Acquisition of T cell data was on BD LSRFortessa, antibody Fc effector functional data on BD LSRII, luminex data on BioPlex200, antibody glycosylation data on Appled Biosystems 3500/3500xL Genetic Analyzer, and microscopy data on Operetta High-Content Imaging Fluorscence Microscope.

Software

FlowJo 10 was used to collect and analyze flow cytometry data. Cell Profiler 3.1.8 was used to quantitate microscopy images.

Cell population abundance

To observe intracellular cytokine staining following antigen stimulation, one million cells/well were plated and stimulated with reported antigens and cocktail as described in materials and methods. A previously published optimized and validated 12 color panel was used to examine cells (De Rosa et al 2012, Horton et al 2007).

Gating strategy

Relevant gating strategies are provided in Supplemental Information.

☒ Tick this box to confirm that a figure exemplifying the gating strategy is provided in the Supplementary Information.
